# Reduced methane emissions in former permafrost soils driven by vegetation and microbial changes following drainage

**DOI:** 10.1111/gcb.16137

**Published:** 2022-03-14

**Authors:** Christoph Keuschnig, Catherine Larose, Mario Rudner, Argus Pesqueda, Stéphane Doleac, Bo Elberling, Robert G. Björk, Leif Klemedtsson, Mats P. Björkman

**Affiliations:** ^1^ Environmental Microbial Genomics Laboratoire Ampere Ecole Centrale de Lyon Ecully France; ^2^ Department of Earth Sciences University of Gothenburg Gothenburg Sweden; ^3^ Ecole Polytechnique Palaiseau France; ^4^ Center for Permafrost (CENPERM) Department of Geosciences and Natural Resource Management University of Copenhagen Copenhagen Denmark; ^5^ Gothenburg Global Biodiversity Centre Gothenburg Sweden; ^6^ Present address: Center for Ecological Research and Forestry Applications (CREAF)‐Edifici C Universitat Autonoma de Barcelona Bellaterra, Barcelona Spain

**Keywords:** Arctic, climate change, methane, post‐permafrost soil, Tundra ecosystems

## Abstract

In Arctic regions, thawing permafrost soils are projected to release 50 to 250 Gt of carbon by 2100. This data is mostly derived from carbon‐rich wetlands, although 71% of this carbon pool is stored in faster‐thawing mineral soils, where ecosystems close to the outer boundaries of permafrost regions are especially vulnerable. Although extensive data exists from currently thawing sites and short‐term thawing experiments, investigations of the long‐term changes following final thaw and co‐occurring drainage are scarce. Here we show ecosystem changes at two comparable tussock tundra sites with distinct permafrost thaw histories, representing 15 and 25 years of natural drainage, that resulted in a 10‐fold decrease in CH_4_ emissions (3.2 ± 2.2 vs. 0.3 ± 0.4 mg C‐CH_4_ m^−2^ day^−1^), while CO_2_ emissions were comparable. These data extend the time perspective from earlier studies based on short‐term experimental drainage. The overall microbial community structures did not differ significantly between sites, although the drier top soils at the most advanced site led to a loss of methanogens and their syntrophic partners in surface layers while the abundance of methanotrophs remained unchanged. The resulting deeper aeration zones likely increased CH_4_ oxidation due to the longer residence time of CH_4_ in the oxidation zone, while the observed loss of aerenchyma plants reduced CH_4_ diffusion from deeper soil layers directly to the atmosphere. Our findings highlight the importance of including hydrological, vegetation and microbial specific responses when studying long‐term effects of climate change on CH_4_ emissions and underscores the need for data from different soil types and thaw histories.

## Graphical abstract

Post‐permafrost changes reduced methane emission from upland mineral soils. Here we show ecosystem changes at two tussock tundra sites with distinct permafrost thaw histories, leading to a 10‐fold decrease in CH_4_ emissions. With progressing time, changes in hydrology reduced water saturation in the topsoil, leading to unfavorable conditions for methanogenesis. Deeper aeration zones likely increased CH_4_ oxidation due to longer residence time in the oxidation zone, while the observed loss of aerenchyma plants reduced CH_4_ diffusion from deeper soil. Our findings highlights the importance of including hydrological, vegetation and microbial responses when studying long‐term permafrost‐thaw effects on potential CH_4_ emissions.

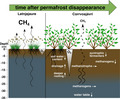

## 1 INTRODUCTION

The northern hemisphere's permafrost soils store an estimated 1.6 Gt of soil organic carbon (SOC), which is double the amount currently measured in our atmosphere (Hugelius et al., 2014; Schuur et al., 2015). With climate warming, this carbon can become available for microbial decomposition resulting in further carbon dioxide (CO_2_) and methane (CH_4_) being released to the atmosphere, thus generating a positive climate warming feedback (Biskaborn et al., 2019; Natali et al., 2021; Schuur et al., 2015, 2021). Our current view on the terrestrial Arctic CH_4_ budget is strongly biased (Margesin & Collins, 2019; Messan et al., 2020; Stackhouse et al., 2017) towards emission hotspots in peat wetlands (Altshuler et al., 2019; Bäckstrand et al., 2010; Lupascu et al., 2012; Mastepanov et al., 2008; Matveev et al., 2018; Merbold et al., 2009; Moguel et al., 2021; Sachs et al., 2008; Sturtevant et al., 2012; Torn & Chapin, 1993; Van Huissteden et al., 2011; Wagner et al., 2005), while less attention has been given to carbon‐poor mineral soils (Emmerton et al., 2014; Jørgensen et al., 2015). These soils, which are subject to much faster thaw rates than peatlands (Westermann et al., 2015), cover ~87% of the region, which corresponds to ~71% of the total estimated SOC within the top 0–3 m (Hugelius et al., 2014; Pérez et al., 2015; Tarnocai et al., 2009). A large proportion of this area is classified as tussock tundra (336,000 km^2^) which has permafrost as a prerequisite for ecosystem functioning (Molau, 2010; Walker et al., 2005). Therefore, any changes to these environments are expected to have significant impacts on the global CH_4_ budget.

Ecosystem soil CH_4_ dynamics are controlled by two co‐occurring microbiological processes; methanogenesis (the production of CH_4_), performed by methanogenic archaea under strictly anoxic conditions in conjunction with fermentative, syntrophic partners; and aerobic methanotrophy (the consumption of CH_4_), performed by methanotrophic bacteria when oxygen is available (Nazaries et al., 2013). Methane_,_ once produced in the soil, can be emitted to the atmosphere through molecular diffusion, ebullition and plant‐mediated transport by aerenchyma plants (Nielsen et al., 2017; Serrano‐Silva et al., 2014). These plants are specialized for flooded areas as O_2_ can diffuse through aerenchyma conduits to anoxic root zones, which in turn allows direct CH_4_ diffusion to the atmosphere, thus bypassing potential CH_4_ oxidation by methanotrophic bacteria. Aerenchyma plants inhabit wetlands, but also intact tussock tundra with permafrost present, where flooded conditions occur frequently due to hampered drainage by the frozen ground (Brix et al., 2001; Greenup et al., 2000; Henneberg et al., 2012; Ström et al., 2003). Tussock forming aerenchyma grasses like the hare's‐tail cottongrass (*Eriophorum vaginatum* L.) thrive under these conditions as their metabolic activity in spring starts early due to their partly aboveground roots (Chapin et al., 1979).

The disappearance of permafrost marks the onset of an ecosystem transition initiated by changes in hydrology (Zona, 2016). Once the frozen soil layer that acts as a barrier to soil drainage disappears, a number of changes have been shown to occur, such as increased topsoil temperatures and aeration (Kwon et al., 2016) which can lead to a shift in vegetation patterns (Bjorkman et al., 2018) including shrubification (Martin et al., 2017). Drainage experiments, carried out in the peatlands of Siberia and Alaska, have shown that these changes are accompanied by a reduction in CH_4_ fluxes (Kwon et al., 2017; Merbold et al., 2009; Sturtevant et al., 2012; Zona et al., 2009) which can be linked to a loss in both methanogenic and methanotrophic microbial communities in the drained soils (Kwon et al., 2017, 2019, 2021). While the overall effect of climate change on drainage and shifts in hydrology is unclear, recent model predictions suggest a long‐term drying of surface soil for permafrost regions due to a larger moisture infiltration to deeper soils (Andresen et al., 2020).

Both emissions and uptake of CH_4_ are sensitive to temperature changes (Le Mer & Roger, 2001; Segers, 1998), and progression/change of seasons (Arndt et al., 2019; Wagner et al., 2003; Zona et al., 2016), with methanogenesis generally responding faster to warming (Segers, 1998). However, high‐affinity methanotrophs, oxidizing CH_4_ at low concentrations, have been suggested to be even more sensitive to temperature changes (Oh et al., 2016, 2020). Furthermore, the type of ecosystem also plays a role when it comes to the relationship between temperature and CH_4_ emissions, with wet tundra (water table at or above soil surface) being more responsive to changes compared to drier sites (water table below soil surface) where the position of the water table impacts CH_4_‐cycling processes (Olefeldt et al., 2013). Thus, the sensitivity of these ecosystems to climatic shifts and permafrost thaw makes them particularly vulnerable to climate changes, regarding both temperatures and hydrological conditions (Elberling et al., 2013; Molau, 2010; Ridefelt et al., 2008; Walker et al., 2006).

Although artificial drainage field experiments are conducted and monitoring of permafrost thaw regions is ongoing (Kwon et al., 2021; Merbold et al., 2009; Schuur et al., 2009; Sturtevant et al., 2012; Zona et al., 2009), more data covering a wider range of Arctic sites, including current and post‐thaw conditions are required in order to better predict the long‐term responses in light of a warming climate (Zona, 2016). To improve our understanding of the long‐term effects of permafrost thaw‐out on CH_4_ emissions, we compared two tussock tundra sites with decadal differences in their complete loss of permafrost. For these upland‐mineral soils, we hypothesized that increased drainage, following the loss of permafrost, will alter the ecosystem's CH_4_ production and emission pathways including: changes in plant composition, with a reduction of wetland associated species, and changes in the microbial community, especially a loss of methanogens in drier soils. This would lead to a reduction in CH_4_ emissions, as the amount of CH_4_ emitted to the atmosphere ultimately depends on the balance between microbial production and oxidation of CH_4_, in combination with changes in the relative importance of emission pathways (Nazaries et al., 2013).

## 2 MATERIALS AND METHODS

### 2.1 Field sites

The two tussock tundra sites used in this study are located in the vicinity of Abisko, in sub‐arctic Sweden, where the presence of tussock tundra communities are restricted to current or former permafrost grounds (Molau, 2010). The site at lake Latnjajaure (68°21.2′N, 18°29.3′E and 981 m a.s.l.) is located close to the Latnjajaure Field Station (LFS), part of the International Tundra Experiment (ITEX; Henry & Molau, 1997) and monitored since 1990. LFS has been experiencing a temperature increase of 0.12°C per year (period 1993 to 2006, (Björk et al., 2007), a trend that has decreased to 0.03°C per year during later years (period 1993 to 2018, Scharn et al., 2021). At this tussock tundra site, the permafrost was last recorded in 1993 and confirmed absent in 2001 (Beylich, 2003; Beylich et al., 2004; Molau, 2010). Although water table depth is not monitored at LFS, the formerly permanently water‐filled boulder pits at the site now drain completely over the growing season (Molau, 2010). The second site, at lake Corrvosjávri (68°24.9′N, 18°38.1′E, and 814 m a.s.l.), was first identified as tussock tundra community via LandSat images and helicopter surveillance in 2005, during a pronounced flowering season of *Eriophorum vaginatum* (Molau, 2010). Here the frozen ground thawed decades ago and the former plant community is now experiencing a transition to a shrub tundra ecosystem, ideal for studying long‐term changes following the warming of active layer and thawing of underlying permafrost. Both sites have a mineral soil identified as Haplic Gleysol, topped by a shallow organic layer (1–8 cm), underlain by a brown (ferric‐containing) mineral soil, followed by a strongly reduced blackish‐grey (ferrous‐containing) mineral soil below 12 cm (Molau, 2010).

### 2.2 Climatic conditions and permafrost

Climate variabilities have been recorded in Abisko since 1913 and reveal a warming event during the late 1930’s and early 1940’s, followed by a colder period (Callaghan et al., 2010). Since the mid‐1970’s, the region is experiencing a warming trend exceeding that of the 1930’s to 1940’s, which also influences permafrost conditions for the region (Johansson et al., 2011). Several approaches were used to predict the occurrence of permafrost for this area, and the likelihood of finding permafrost around Latnjajaure is estimated to be <50% (Gisnås et al., 2017; Ridefelt et al., 2008). Corrvosjávri falls outside the predictive area for these local high resolution modelling attempts but is located within the area covered by regional models that estimate the sporadic occurrence of permafrost. Since fine‐scale reconstructions of historical permafrost distribution are rare for the Abisko region and surrounding areas (Yang et al., 2012), we used temperature data from LFS and Abisko Scientific Research Station to estimate a time window of permafrost thaw at Corrvosjavri. Environmental lapse rates were established on a monthly basis using daily average temperatures (period 1993–2019) from the automatic weather station at LFS (Björk et al., 2007) and the meteorological observations at Abisko Scientific Research Station (Callaghan et al., 2010 ‐ *Data available from the Swedish Polar Research Secretariat* 1). The calculated monthly lapse rates are in line with earlier observations from the region (Table S2) and were used to model the Mean Annual Air Temperatures (MAAT) at both Latnjajaure and Corrvosjavri based on the historical record from Abisko (1913–2019, Figure 1). Furthermore, the MAAT was smoothed using a 5‐year running mean according to (Yang et al., 2012) and a polynomial fit was established for the entire period (Callaghan et al., 2010).

**FIGURE 1:**
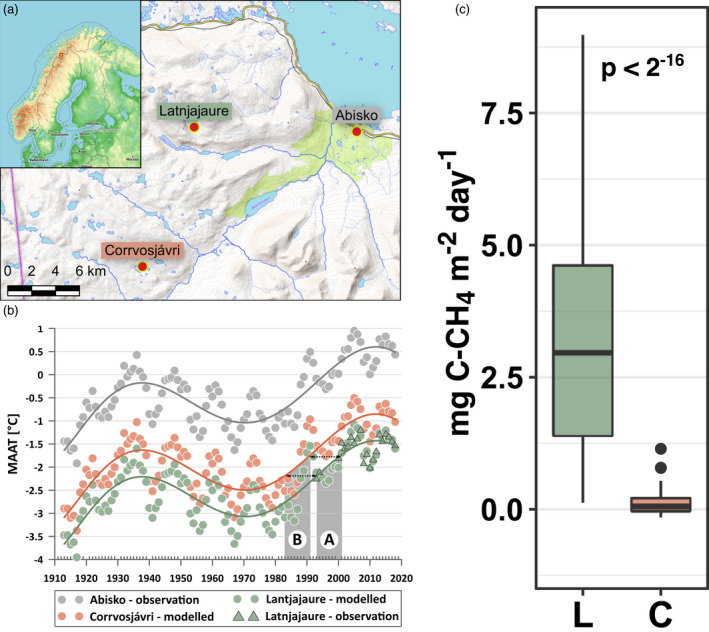
Location of the two studied field sites in northern Sweden (a), Mean Annual Air Temperatures ‐ MAAT (b) and measured CH_4_ emissions at the two sites (c). b: MAAT was smoothed to a 5‐year running mean according to Yang et al. (2012) at Abisko, based on the long‐term historical record from Abisko Scientific Research Station (Abisko – observation, 1913–2019). Historical data was modelled for Latnjajaure and Corrvosjávri based on monthly lapse rates (Table S2) established from the over 20 years of overlapping datasets between Abisko and Latnjajaure Field Station (LFS). A polynomial function was imposed on each dataset as described by Callaghan et al. (2010). The shaded area “A” indicates the time‐window when permafrost was last observed (1993) and confirmed absent (2001) in Latnjajaure (Beylich, 2003; Beylich et al., 2004; Molau, 2010). The shaded area “B” indicates the corresponding time‐window for when Corrvosjávri passed the same threshold based on the modelled temperature data. Included is also the observed temperature record from LSF, which fit the predicted MAAT values from the model. c: Methane emissions over the growing season 2016 to 2018 at the two studied tussock tundra sites Latnjajaure (L) and Corrvosjávri (C); *n* = 54. The difference between group means per study site was tested by a Wilcoxon rank‐sum test

Given that permafrost was confirmed present at Latnjajaure 1993, and confirmed absent to a depth of at least 40 m in 2001 (Beylich, 2003; Beylich et al., 2004; Molau, 2010), the final thaw coincides with the period when the long‐term MAAT reaches >2°C, a threshold that was previously suggested as the lower limit for sporadic permafrost occurrence in the Scandes (Ødegård et al., 1996). Corrvosjavri passed this threshold during the 1980’s (Figure 1). This results in a final permafrost thaw difference of a minimum of one decade between the two sites. However, different threshold MAATs have been estimated for mountainous regions (Etzelmüller et al., 2006; Haeberli et al., 2011), and local variations in topography, snow cover and aspect, among others (Johansson et al., 2006), may also influence the actual soil temperatures and permafrost conditions. There is a lack of historical data from the two sites, especially Corrvosjávri which lies outside the commonly investigated area around Abisko and LFS. Corrvosjávri might have lost its permafrost earlier than estimated here, and likely had a phase during the 1930’s to 1940’s warm period with less favorable permafrost conditions.

### 2.3 Vegetation

The vegetation at both sites was surveyed during mid‐July in both 2006 (Molau, 2010) and 2016 using the point‐intercept methodology (Molau & Mølgaard, 1996) with 20 point‐frame squares (0.25 m^2^ and 25 intercepts each) laid out as two transects (20 m each) in a cross and a 2 m distance between each point‐frame location. The 2016 transects were laid out within a couple of meters of the 2006 transects based on photographs. The overall trend in vegetation was analyzed using Non‐metric multidimensional scaling (NMDS) with Bray‐Curtis distances (Canoco 5 software) where the influence of single occurring plants was down‐weighted.

### 2.4 Flux and environmental measurements

During the growing season of 2016–2018, flux measurements of CH_4_ and CO_2_ were conducted on a bi‐weekly basis at both sites, from early June to the end of August with an additional campaign in early October 2017 (a total of 20 field campaigns), utilizing a closed chamber technique on pre‐installed soil collars. Three groups of three collars were installed at each site within the area covered by the vegetation surveys, and gas flux data were averaged for each group (*n* = 3 per site). Fluxes of CH_4_ and CO_2_ fluxes were measured using an ultraportable greenhouse gas analyzer (Los Gatos Research) operating at 1 Hz, with a precision of <2 ppm and <300 ppm (respectively) and an operational range of 0–500 and 0–20,000 ppm, respectively. For net exchanges of CH_4_ and CO_2_, the analyzer was connected to a transparent chamber (*r* = 9.5 cm, *h* = 20 cm), while ecosystem respiration was measured in darkness by covering the chamber with an additional opaque hood during measurements. Both net fluxes and respiration rates were measured three times each from every collar, with a 5 min closure time and one‐minute aeration of the system in between measurements. The change in chamber concentration over time was then calculated using linear regression of the dry concentration of each gas (provided by the instrument), disregarding the first 30s of all measurements and regression with *R* ^2^ < .8 for the ecosystem respiration. No threshold cutoff was made for the net exchange of CO_2_ and CH_4_ since these can be both positive, negative or zero (giving low *R* ^2^ values).

Furthermore, soil temperature (‐5 cm) and soil moisture were measured four times around each collar using handheld soil thermometers and a Delta ML2x Theta probe (Delta‐T Devices Ltd), respectively. Meteorological parameters such as air temperature, snow depth, precipitation, and pressure were recorded using a U30 HOBO automatic weather station (Onset Computer Corporation, Bourne, Massachusetts, U.S.) at each site, and soil temperatures were logged at 2, 10 and 30 cm depths at two locations in each site (TinyTag, Gemini Data Loggers). Measurements from the local weather station at Latnjajaure Field Station (Scharn,, Brachmann, et al., Scharn Little^2021^, ^2021^) showed that the 2016 and 2017 growing season air temperatures were within the average, with July temperatures 9.3 ± 4.0 and 7.3 ± 4.3°C, respectively (July average 1993–2019: 8.6 ± 4.1°C), while the growing season of 2018 was warmer, with average July temperatures at 12.7 ± 4.9°C. The seasonal precipitation at the most representative meteorological station Katterjåkk was 243, 216 and 352 mm for the three years 2016 – 2018 (JJA average 1993–2019: 222 ± 66 mm) (Scharn et al., 2021).

Seasonal fluxes of CH_4_ (July–August) were established for each collar set using interpolation (Table S1). Classic first‐order exponential models between CH_4_ emissions and temperature (D’Imperio et al., 2017; Grogan & Jonasson, 2005), Temperature sensitivity coefficients (Q_10_) and Activation Energy (*E_a_*) were established for each group of three collars, using emission data with *R* ^2^ > .8 (Table S1). Q_10_ and *E_a_* were estimated based on the Arrhenius equation, by plotting the natural logarithm of in situ CH_4_ emissions against the measured soil temperature (in 1000/K) (Davidson & Janssens, 2006). For comparison, seasonal fluxes were also calculated using the exponential models and the hourly data from the Tiny Tag loggers at 2 cm depth, using the same period as for the interpolated data (Table S3).

### 2.5 Soil sampling

Soil samples for microbial and chemical analysis were collected <0.6 m from each collar used for gas flux measurements. The top organic layers (0–5 cm) and deep mineral layers (15–30 cm) of the tussock soils were sampled using either an electric drill (*r* = 1 cm), during the initial frozen conditions, or with a soil auger (*r* = 1.5 cm). Soil samples were pooled to form one homogenized sample per soil depth for each group of three collars, allowing for comparisons with the flux data (*n* = 3 per site and soil layer). Microbial samples were transferred to sampling tubes with silica gel (both sterilized), samples for chemical analysis were collected in Ziploc bags, and both were stored cold during fieldwork and frozen within one week of sampling. Microbial samples were shipped frozen to the University of Lyon, France, while biochemical samples were shipped to the University of Gothenburg, Sweden.

### 2.6 Soil physical and chemical measurements

Gravimetric soil water content was carried out by drying soil at 70°C for 48 h, and soil organic matter (SOM) was measured as a loss on ignition by heating the dried samples at 550°C for 6 h. Parts of the dried samples were also grinded followed by C and N quantification using Isotope Ratio Mass Spectrometry (HS2022, Sercon Limited). The pH was measured by shaking dry soil with deionized water (ration 1:10) for half an hour and pH was then recorded (691 pH Meter, Metrohm, Riverview, Florida, U.S.) after sedimentation overnight. The procedure was repeated with the addition of 1 M KCl to a final concentration of 0.1 M in the solution.

### 2.7 Microbial community analysis

DNA was extracted from 0.25 to 0.5 grams of soil using a protocol that has been successfully applied on various soil types including often difficult to extract clay soils (Griffiths et al., 2000). DNA was subsequently fluorometrically quantified (Qubit, Invitrogen^TM^), diluted to a concentration of 1 ng µl^−1^ and stored at −20°C until experimental use. Targeted amplicons were generated with a Platinum Taq (Invitrogen^TM^) assay for the PCR step using modified 515f/806r (Walters et al., 2015), MLf/MLr (Luton et al., 2002) and A189f/A650r (McDonald et al., 2008) primer pairs with the Nextera Illumina adapter sequences (5′‐TCGTCGGCAGCGTCAGATGTGTATAAGAGACAG‐3′ and 5′‐GTCTCGTGGGCTCGGAGATGTGTATAAGAGACAG‐3′) attached to the 5′ end to amplify the V4 region of the 16S rRNA gene, methyl coenzyme‐M reductase subunit A (*mcrA*) and particulate methane monooxygenase (*pMMO*) respectively. PCR reactions (25 µl final volume) were set up as recommended in the supplier protocol with 5 to 10 ng of DNA, 0.5 µl of the primer pair (10 µM) and 200 ng of UltraPure BSA (Invitrogen^TM^). The temperature program for the reaction was 3 min at 94°C followed by 30 (*16S rRNA*), 40 (*mcrA*) and 40 (*pmoA*) cycles of 30 s at 94°C, 30 s at 50°C (*16S rRNA*), and 55°C (*mcrA* and *pmoA*) and 60 s at 72°C followed by 10 min at 72°C and subsequent 4°C to stop the reaction. Successful amplifications of the targeted gene amplicons were confirmed on agarose gels and only assays with no amplification in the negative control (ultrapure water used during DNA extraction and library preparation) were further processed. Amplified DNA was cleaned‐up using AMPure XP Beads (Beckman Coulter^TM^) and used for the subsequent PCR with Illumina Nextera XT index primers to add barcode sequences to amplicons of each sample followed by another round of bead clean‐up. The concentration of resulting libraries of each sample was measured spectrophotometrically and subsequently combined to 3 equimolar pools, each representing libraries from one targeted gene. The pools were run on a DNA 1000 chip (Agilent 2100 Bioanalyzer) to verify absence of primer dimers and the correct size of the libraries. Molarities of final pools were estimated by quantitative PCR using the QuantiFast SYBR^®^ PCR Kit (Qiagen) with primers targeting the P5 and P7 flanking regions of Illumina Nextera XT libraries with a standard assay and two‐step cycling program recommended by the supplier. Dilution series of successfully sequenced libraries were used as standards. Pools were diluted to 4 nM and loaded on a V2 2 × 250 bp flow cell for paired‐end sequencing on an Illumina MiSeq platform following the protocol provided by Illumina.

### 2.8 Sequence processing

Forward and reverse read files for all sequenced genes of each sample were merged using the *iu*‐*merge*‐*pairs* command from the Illumina‐Utils v2.6 libraries using the—*enfore*‐*Q30*‐*check* flag to ensure quality filtering over the entire read length before merging (Eren et al., 2013). Successfully merged amplicons of the V4 region of the *16S rRNA* gene were filtered for chimeric sequences and further annotated with the RDP classifier (Edgar et al., 2011; Wang et al., 2007). Taxonomic annotation of the sequenced marker genes was performed by placing sequenced amplicons in a reference tree using GraftM (version v0.13.1; https://github.com/geronimp/graftM—released under GNU General Public License v3+) (Boyd et al., 2018). The graftM package including reference sequences for *mcrA* gene was downloaded from the repository at GitHub. For *pmoA* annotation, a multiple sequence alignment calculated with MAFFT (version v7.475) (Katoh et al., 2002) of 7809 sequences downloaded from the *pmoA* gene reference database at the GFZ Potsdam (Yang et al., 2016) was used to create a graftM package. All further processing and analysis on annotation tables were carried out using the R‐package *phyloseq* (McMurdie & Holmes, 2013; Core R Team, 2019). Significances of differences of means between sample groups were estimated by the *ggpubr* package. Differential abundance analysis on the *16S rRNA* gene dataset was performed using the *DESeq2* package. Diversity estimates were calculated with the R‐packages *vegan* and *betadisper*.

### 2.9 Marker gene quantification of methane cyclers

Primer pairs 341F/534R (Watanabe et al., 2001), MLf/MLr (Luton et al., 2002) and A189f/A650r (McDonald et al., 2008) targeting the *16S rRNA*, *mcrA* and *pmoA* gene respectively were used to estimate gene copy abundances in DNA samples by quantitative PCR (QuantiFast SYBR^®^ PCR Kit; Qiagen). For standards, amplicons of the respective genes were amplified from a soil sample, inserted in a pGEM‐T vector (Promega) and cloned in chemically competent *E*.*coli* cells (Invitrogen^TM^) spread out on selective LB‐agar. PCR with M13 primers flanking the insert site on the vector on successfully cloned colonies was sent for Sanger sequencing to confirm correct sequences of the targeted genes (Eurofins Genomics). M13 amplicons of correct sequences were quantified (n=3) by a Qubit assay (Invitrogen^TM^) for estimation of standard copy numbers. Gene quantification assays contained 1–5 ng of DNA, 0.5, 1.4 and 1.4 µM of respective primers for *16S rRNA*, *mcrA* and *pmoA* genes, 200 ng of UltraPure BSA (Invitrogen^TM^), 10 µL of the 2x master mix and ultra‐pure water (final volume 20 µL). A two‐step cycling program with 5 min at 95°C followed by 30, 35 and 35 cycles (*16S rRNA*, *mcrA* and *pmoA*, respectively) of 5 sec at 95°C and 30 s at 60°C finished by a melting curve (60–98°C; 1°C /min increase) was performed in a Corbett Rotor‐Gene 6000 real‐time PCR cycler. Each run included standards of the respective targeted gene in a range of 10^1^–10^7^ copies/reaction as well as non‐template controls to check for contamination. Runs with no amplification in the no‐template‐control and values >0.98 and between 0.9 and 1.1 for *R* ^2^ and efficiency from the standard curves were used for further analysis and plotting in *R*.

## 3 RESULTS

### 3.1 CH_4_, CO_2_ emissions and soil geochemistry

**FIGURE 2:**
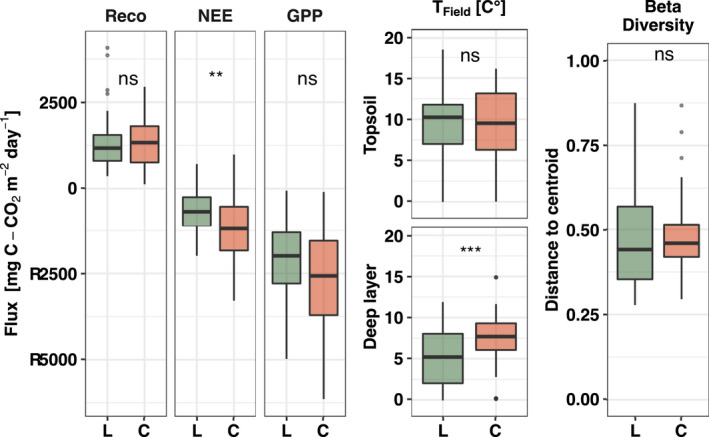
Ecosystem Respiration (Reco), Net Ecosystem Exchange (NEE), Gross Primary Production (GPP); soil temperature (*T*_Field_) at 2 cm and 30 cm depth (topsoil and deep layer, respectively) from the bi‐weekly measurements during the growing seasons 2016–2018, and the microbial beta‐diversity based on the Bray‐Curtis dissimilarity matrix of annotated bacterial and archaeal *16S rRNA* amplicon sequences from the bi‐weekly sampling 2017 at Latnjajaure (L) and Corrvosjávri (C) in top soil and deeper soil layers

Measured CH_4_ emissions at the study sites with decadal differences in their permafrost history decreased 10‐fold. Latnjajaure (loss of permafrost between 1993 and 2001) showed emissions of 3.9 ± 3.2 mg C‐CH_4_ m^−2^ day^−1^ (growing seasons 2016–2018), while Corrvosjávri (estimated loss of permafrost in the 1980s based on MAAT records) showed 0.2 ± 0.3 mg C–CH_4_ m^−2^ day^−1^ (growing seasons 2016–2018, Figure 1), with similar magnitude for the seasonal flux (Table S1). Based on the three years of monitoring data, our two sites had comparable surface soil temperatures, ecosystem respiration, and gross primary productivity, but deeper soil temperatures and net ecosystem uptake rates were significantly higher at the site with longer post permafrost progression (Figure 2). Furthermore, higher pH was measured at both top and deeper soil layers in Corrvosjávri (Figure S1). Soil water content (SWC), soil organic matter (SOM) and total carbon were found to be significantly higher in Latnjajaure topsoils, while in deeper layers only total carbon and nitrogen (and resulting C/N ratios) were found to be higher at this site (Figure S1).

### 3.2 Vegetation cover

**FIGURE 3:**
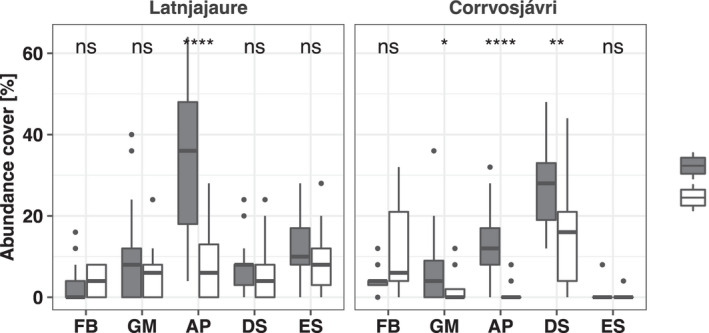
Plant community counts of distinct classes as abundance cover at Latnjajaure and Corrvosjávri surveyed in 2006 and 2016 including Forbs (FB), Graminoids (GM), Aerenchyma Plants (AP), Deciduous Shrubs (DS), and Evergreen Shrubs (ES)

**FIGURE 4:**
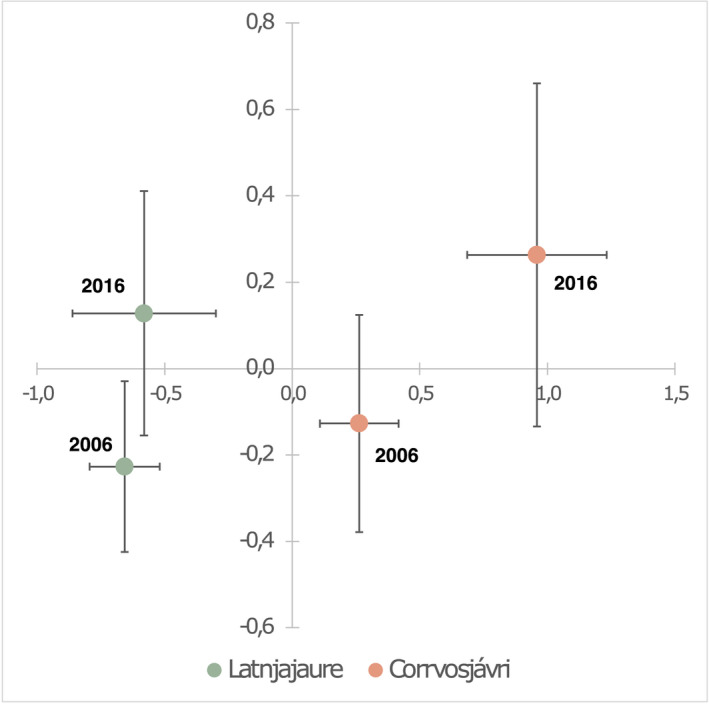
Non‐metric multidimensional scaling (NMDS) with Bray‐Curtis distances of vegetation cover data showing the overall trend between 2006 and 2016 for Latnjajaure and Corrvosjávri

Plant cover for forbs, graminoids, aerenchyma plants, deciduous shrubs and evergreen shrubs was recorded in 2006 and 2016 at the two study sites (Figure 3), deviating in their species composition but indicating similar development trajectories through time (Figure 4). A significant loss of aerenchyma plants was observed during this time at both sites (Figure 3). While still present at Latnjajaure in 2016, aerenchymatous plants had almost completely disappeared at Corrvosjávri, with only a few datapoints confirming their presence at low abundance cover (<10%). At Corrvosjávri, graminoids and deciduous shrubs also showed a significant decrease during this timeframe (Figure 3).

### 3.3 Soil microbial communities

The overall bacterial and archaeal community structure, based on sequenced 16S rRNA amplicons, was comparable between the two sites in both top and deeper soil layers (Figure 2 and Figure S2).

**FIGURE 5:**
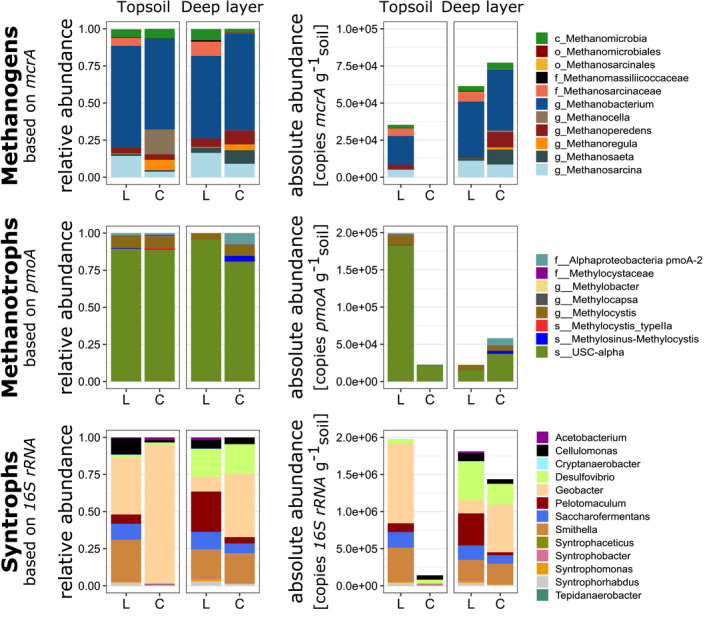
Relative and absolute abundance for methanogenic, methanotrophic and potential syntrophic communities identified in two sampled soil layers (topsoil and deep layer =0 to −5 and −15 to −30 cm respectively) at the two studied tussock tundra sites Latnjajaure (L) and Corrvosjávri (C) over the growing season in 2017. Phylogenetic levels of successful annotations of sequenced *mcrA* and *pmoA* amplicons placed in a reference tree by GraftM are indicated before the taxonomic name in the form of “phylogenetic level_taxonomic name” (with c, o, f, g, s referring to class, order, family, genus and species, respectively). Syntrophic taxa were filtered from sequenced *16S rRNA* amplicons by a set of potential syntrophic genera identified in the literature. Absolute abundances were calculated by multiplying relative abundance with the copy number estimated by qPCR of the respective sample

**FIGURE 6:**
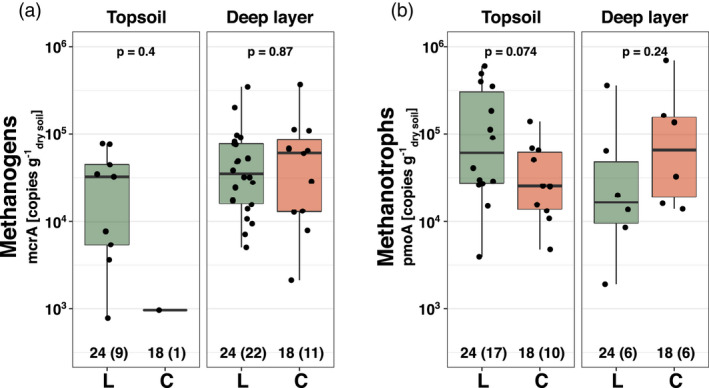
Absolute abundance of the methanogenic (a) and methanotrophic (b) communities estimated by *mcrA* and *pmoA* gene copies respectively in topsoil (0 to −5 cm) and deep layer (−15 to −30 cm) samples of the two study sites (Latnjajaure (L); Corrvosjávri (C)). Differences between group means per study site were tested by a Wilcoxon rank‐sum test. Numbers at the bottom of the graph represent the number of total observations per group and the number of observations above the detection limit in brackets

Methanogenic communities, based on sequenced *mcrA* amplicons, were found to be similar at both sites and soil depth based on relative abundance with *Methanobacterium* as the dominating genus (between 55% and 68% in relative abundance) followed by *Methanosarcina* (Figure 5). However, the absolute abundance of *mcrA* gene copies estimated by qPCR showed a near‐complete absence of methanogens in the topsoil of Corrvosjávri (9.6 × 10^2^ copies *g*_dry soil_^−1^; detected in one out of 18 replicates), while the topsoil at Latnjajaure held similar abundances (mean of 3.5 × 10^4^ copies g_dry soil_^−1^; detected in 9 out of 24 replicates) as the deep layers of both sites (mean of 6.2 × 10^4^ and 7.7 × 10^4^ copies g_dry soil_^−1^; detected in 22 out of 24 and 11 out of 18 replicates at Latnjajaure and Corrvosjávri, respectively) where no significant difference in *mcrA* abundance was observed (Figure 6).

There were also no significant differences between the methanotrophic communities of both sites based on sequenced and quantified *pmoA* genes (Figure 5). Relative abundances showed a similar community structure dominated by sequences annotated to the upland soil cluster alpha (USC—alpha; between 76% and 96% in relative abundance) followed by the genus *Methylocystis*. Abundance estimates of *pmoA* gene copies showed no significant difference between the two sites (Figure 6).

Genera known to act as syntrophic partners in methanogenesis were extracted from total sequenced bacterial *16S rRNA* gene amplicons showing presence of *Geobacter*, *Smithella*, *Desulfovibrio* and *Pelotomaculum* related sequences among others (Figure 5). When absolute abundances of these taxa were estimated with data from quantitative PCR on bacterial *16S rRNA* genes, a drop in abundance for syntrophic partners in the topsoil of Corrvosjávri, similar to the results of the *mcrA* gene abundance, was observed (Figure 5). Following these results, a differential abundance analysis on the total 1150 identified bacterial and archaeal taxa was used to identify those who significantly differ in abundance between the two studied sites. This resulted in a list of 16 facultative or obligate anaerobic taxa (threshold log_2_ fold change = 2) with significantly higher abundances at Latnjajaure, of which 9 have been shown to act as syntrophic organisms in methanogenesis and 3 were methanogens (Table S2). In contrast, nine taxa found to be higher abundant in Corrvosjávri were linked to aerobic lifestyles. The identified differences in community structure are in line with the qPCR results showing lower methanogen and syntrophic organism abundances in topsoil of Corrvosjávri compared to Latnjajaure, as described above.

## 4 DISCUSSION

### 4.1 Impact of drainage on CH_4_ emissions

Here we show how long‐term ecosystem change alters CH_4_ cycling using a space for time approach (Blois et al., 2013) on two field sites (space) with decadal differences (time) in the permafrost thaw histories (Figure 1). The two sites represent differential natural drainage regimes and can be used to test the long‐term effects of climate change‐induced shifts in hydrology in natural tundra soils (Molau, 2010). Measured CH_4_ emissions decreased 10‐fold with the longer absence of permafrost. These fluxes are allocated in the lower end of earlier observations from permafrost tussock tundra ecosystem, typically ranging from slight uptakes (−1.5 to 0 mg C–CH_4_ m^−2^ day^−1^, e.g, Blanc‐Betes et al., 2016; Kwon et al., 2017; Whalen & Reeburgh, 1990), to low (0 to 100 mg C–CH_4_ m^−2^ day^−1^, e.g. (Oberbauer et al., 1998; Sturtevant et al., 2012; Torn & Chapin, 1993; Zona et al., 2009)), to high and extreme (100 to >1000 mg C–CH_4_ m^−2^ day^−1^, e.g., Christensen et al., 2000; Corradi et al., 2005; Kwon et al., 2017; Merbold et al., 2009; Mastepanov et al., 2008) emissions, determined by water saturation level, carbon content, plant composition and climatic conditions. Reduction in CH_4_ emissions, similar to ours, have also been observed in experimentally drained sites (Kwon et al., 2017) and were attributed to a shift in vegetation and a loss in both methanotrophic and methanogenic communities in topsoils. Our results on soil temperature and CO_2_ emissions contrast findings from experimental drainage studies in organic‐rich flood plains (Kwon et al., 2016, 2021; Merbold et al., 2009), that show warmer topsoils, reduced CO_2_ emissions, higher respiration and decreased gross primary production following drainage. In these studies, the increase in topsoil temperatures was attributed to a loss in heat capacity and thermal conductivity, which also may explain the contrasting CO_2_ fluxes (Kwon et al., 2016). In mineral soils, like ours, where the mineral layer is overlaid by a shallow organic surface layer, heat transfer differs due to the contrasting soil profiles properties. In addition, drainage was shown to occur over time in the topsoil (Figure S1) but did not affect water content significantly in the deeper soil mineral layers. Although the deeper soil layers at Corrvosjávri had higher temperatures, they did not contribute to significantly higher respiration. The generally observed lower microbial abundance in deeper soil layers at both sites (Figure S2) supports such a scenario with respiration rates dominated by surface soils. In addition, the overall bacterial and archaeal communities were not significantly different between sites (Figure 2 and Figure S2), indicating similar respiratory capacities.

### 4.2 Impact of vegetation shifts on CH_4_ emissions

The observed loss of aerenchyma plants at both sites is likely due to changes in the topsoil water conditions (Figure 3 and Figure S1). This trend is in line with previous long‐term observations at Latnjajaure (1995–2016, Figure S3) (Molau 2012; Scharn et al., 2021, 2021). Aerenchyma plants have a competitive advantage in flooded soil due to their ability to transport O_2_ from the atmosphere to anoxic root zones, an advantage which is lost once the soil is drained (Iversen et al., 2015). Therefore, the observed higher water content in the Latnjajaure topsoil might still support aerenchyma plants, while the dryer topsoil at Corrvosjávri led to a near to complete loss of these plants over the same period. Furthermore, the increased net ecosystem uptake rates observed at Corrvosjávri (Figure 2) are likely linked to a change in vegetation cover (Mekonnen et al., 2018) between the two sites (Figure 4), with an increased occurrence of deciduous shrubs and forbs at this site compared to Latnjajaure (Figure 3).

### 4.3 Impact of microbial community composition on CH_4_ emissions

Despite the differences in observed CH_4_ emissions between Latnjajaure and Corrvosjávri, the overall structure of both methanogenic and methanotrophic communities was similar at both sites and soil layers (Figure 5). This contrasts to experiments conducted in the laboratory, where strong shifts in the microbial community were reported when permafrost was thawed in controlled conditions (Coolen & Orsi, 2015; Wei et al., 2018; Yang et al., 2017). Such experiments are generally conducted over shorter time scales and are difficult to compare to direct measurements in our field sites, as field sites have slowly progressed towards non‐permafrost conditions over the past 40–50 years. This included natural variabilities in climate conditions and soil water content over seasons and years, smoothening any abrupt changes and thereby leading to the development and maintenance of comparable microbial communities. It is likely that the significant changes in community composition, similar to observations in laboratory studies, occurred just after permafrost thaw and drainage, which we were unable to capture in this study. Methanotophs belonging to the *USC*‐alpha dominated the CH_4_ oxidizing community, a clade ubiquitous in soil describing high‐affinity methanotrophs able to metabolize CH_4_ at atmospheric concentrations (Holmes et al., 1999; Kolb, 2009; Lau et al., 2015). Abundances of the CH_4_ oxidizing community were found in similar levels in both sites and soil layers, while CH_4_ producing microbes were lost from topsoil with ongoing time after permafrost disappearance. Altogether, the results on the CH_4_ transforming microbiome suggest that while the overall potential for CH_4_ oxidation is unaffected over time, CH_4_ production in drier surface soil layers cannot be maintained over time due to a reduction of the methanogenic community.

### 4.4 Conceptual framework

**FIGURE 7:**
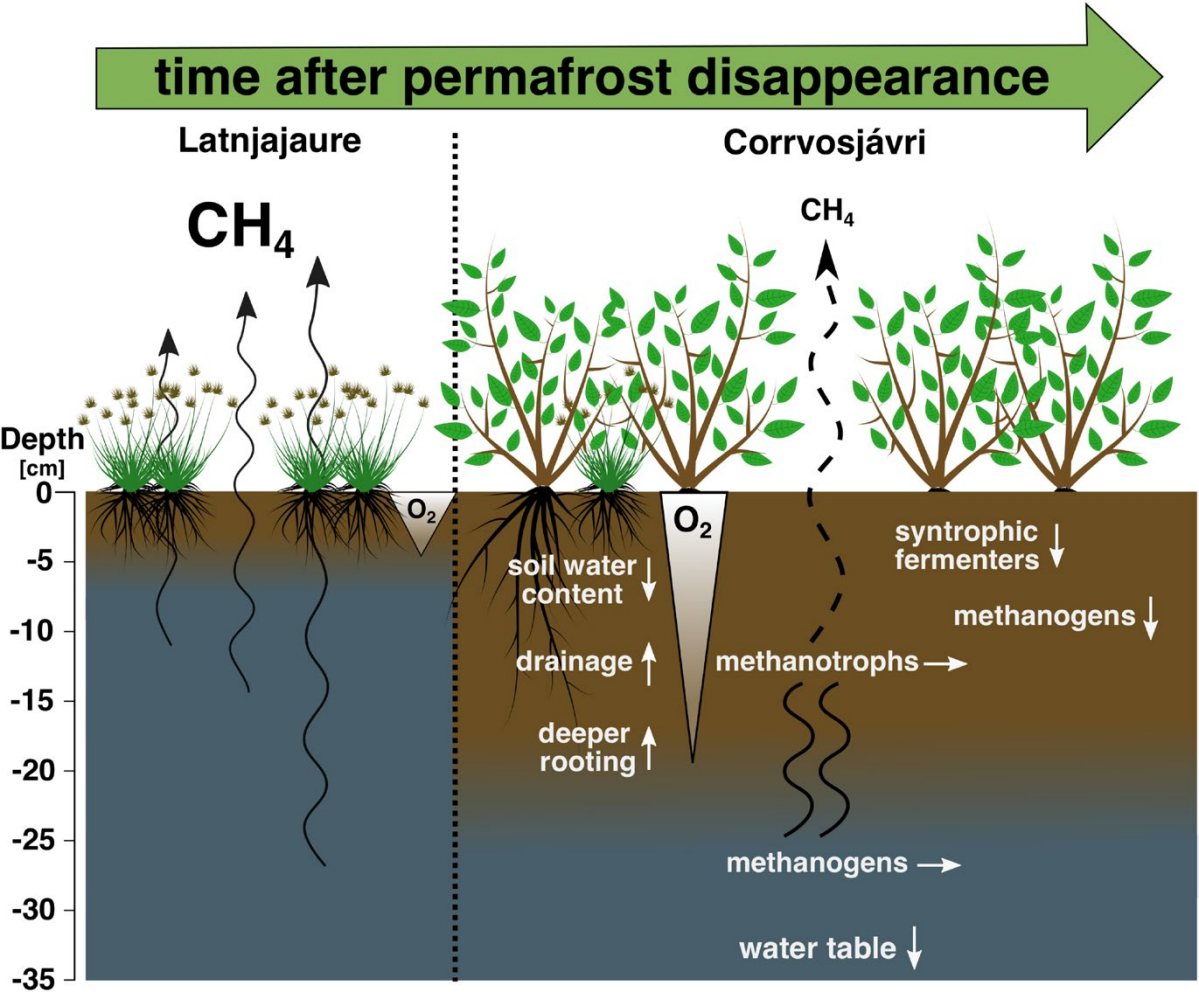
Schematic overview of ecosystem changes that influence CH_4_ emissions in Tussock tundra soils with an estimated decade difference in permafrost disappearance. Arrows in the graph indicate increase (upward arrow), decrease (downward arrow) and no change (horizontal arrow) in parameters. With progressing time, the water table drops and changes the overall hydrology, reduces water saturation and increases oxygen availability in the topsoil layers. These conditions are unfavorable for methanogenesis, leading to a decrease in the abundance of methanogens and their syntrophic partners. Aerenchyma plants that mediate CH_4_ diffusion from deeper soil through their spongy tissue, are replaced by deeper rooting shrubs found in dryer ecosystems. The change in plant species composition and dryer surface soils leads to reduced transport of CH_4_ by ebullition and through plants, thus increasing the residence time of CH_4_ within the soil profile. This enhances the fractio of CH_4_ nsumed by methanotrophs in the oxic zone

The marked decrease of potential CH_4_ producing organisms in the topsoil decades after permafrost thaw suggests that conditions had become unfavorable for methanogenesis in the Corrvosjávri surface layer, which has also previously been observed in drying experiments (Kwon et al., 2017, 2021). Methanogenesis occurs in strict anoxic conditions, for example in water‐saturated soils such as swamps, fens, deeper soil layers, and anoxic micro aggregates in the soil (Bengtson et al., 2012; Le Mer & Roger, 2001; Serrano‐Silva et al., 2014; Watanabe et al., 2007). Increased O_2_ levels and availability of other electron acceptors, which provide a higher redox potential than CO_2_, have been shown to inhibit CH_4_ production (Dalal et al., 2008). A decrease in soil water content in the topsoil (Figure S1), was measured across our thaw gradient, pointing towards increased drainage, or potentially a combination of drainage and increased evapotranspiration (Bring et al., 2016). Drier topsoil also facilitates O_2_ diffusion from the atmosphere into deeper soil layers (Gebauer et al., 1996; Lawrence et al., 2015; Wilson et al., 1985) that consequently inhibits methanogenic activity (Figure 7). In addition, a lower water table favours shrubs over aerenchyma plants (Kwon et al., 2016), thereby reducing plant‐mediated CH_4_ transport (Figure 7). Increased root biomass (Björk et al., 2007) might further impact drainage by the creation of macropores and increase evapotranspiration potential (Angers & Caron, 1998; Fan et al., 2017). As methanogenesis is a syntrophic process depending on fermenting bacteria that provide precursors for CH_4_ formation by the breakdown of complex organic matter, we hypothesized that a shift in members of this community would also have occurred. Results from a differential abundance analysis on the total microbial community supports such a scenario as almost all taxa found to be significantly higher abundant in Latnjajaure were found to be linked to methane production (Table S2). This is striking, as the observed decrease in emitted CH_4_ is linked to a change of a very small fraction of the microbiome (16 out of 1150 taxa), while the remaining microbial community seems to follow the same progression pattern at both sites and does not change anymore with ongoing time. Taken together, our data suggest that anaerobic processes become restricted to deeper soil layers over time in former permafrost mineral soils (Figure 7).

## 5 CONCLUSION

In conclusion, the observed changes in soil properties, plant cover and microbial communities will reduce CH_4_ emissions in former permafrost soils by: (a) restricting CH_4_ production in surface soil as a result of increased oxygen availability in drained and dryer surface soils, (b) reducing plant‐mediated CH_4_ transport (diffusion) via aerenchyma tissue as a result of unfavorable growing conditions for sedges and rushes and the introduction of taller shrubs, and (c) limiting CH_4_ emissions from deeper soil horizons due to increased oxidation by methanotrophs as a result of longer CH_4_ diffusion time through the aerated surface soil.

The mechanistic explanation provided here highlights the importance of accurately estimating soil drainage conditions and plant‐mediated effects on surface soil processes when assessing climate feedbacks from the Arctic. Areas with a topographic potential for drainage would thus reduce their CH_4_ emissions to almost zero over decadal scales after final permafrost thaw, which is in line with previous findings (Bansal et al., 2016; Kwon et al., 2021; Whalen & Reeburgh, 1990). This is to date not accounted for in global climate models. Our results highlight the need for more long‐term, field‐based approaches when it comes to climate‐driven changes in drainage conditions not the least in relation to permafrost‐affected ecosystems.

## AUTHOR CONTRIBUTIONS

C.K. and C.L. conducted analysis and wrote the manuscript. M.R., A.P., S.D. obtained field data and/or conducted analysis. B.E., L.K. and R.G.B. contributed to research design and data interpretation. M.P.B. designed the research, obtained field data, conducted analysis and wrote the manuscript. All authors discussed the study results and reviewed the manuscript.

## CONFLICT OF INTEREST

The authors declare no conflict of interest.

## Supporting information

Supplementary Material

## Footnotes

^1^

https://www.polar.se/en/research‐support/abisko‐scientific‐research‐station/weather‐data/

## References

[gcb16137-bib-0001] Altshuler, I. , Hamel, J. , Turney, S. , Magnuson, E. , Lévesque, R. , Greer, C. W. , & Whyte, L. G. (2019). Species interactions and distinct microbial communities in high Arctic permafrost affected cryosols are associated with the CH_4_ and CO_2_ gas fluxes. Environmental Microbiology, 21(10), 3711–3727. 10.1111/1462-2920.14715 31206918

[gcb16137-bib-0002] Andresen, C. G. , Lawrence, D. M. , Wilson, C. J. , McGuire, A. D. , Koven, C. , Schaefer, K. , Jafarov, E. , Peng, S. , Chen, X. , Gouttevin, I. , Burke, E. , Chadburn, S. , Ji, D. , Chen, G. , Hayes, D. , & Zhang, W. (2020). Soil moisture and hydrology projections of the permafrost region‐a model intercomparison. Cryosphere, 14(2), 445–459. 10.5194/tc-14-445-2020

[gcb16137-bib-0003] Angers, D. A. , & Caron, J. (1998). Plant‐induced changes in soil structure: processes and feedbacks. Biogeochemistry, 42, 55–72.

[gcb16137-bib-0004] Arndt, K. A. , Oechel, W. C. , Goodrich, J. P. , Bailey, B. A. , Kalhori, A. , Hashemi, J. , Sweeney, C. , & Zona, D. (2019). Sensitivity of methane emissions to later soil freezing in Arctic tundra ecosystems. Journal of Geophysical Research: Biogeosciences, 124(8), 2595–2609. 10.1029/2019JG005242

[gcb16137-bib-0005] Bäckstrand, K. , Crill, P. M. , Jackowicz‐Korczyñski, M. , Mastepanov, M. , Christensen, T. R. , & Bastviken, D. (2010). Annual carbon gas budget for a subarctic peatland, Northern Sweden. Biogeosciences, 7(1), 95–108. 10.5194/bg-7-95-2010

[gcb16137-bib-0006] Bansal, S. , Tangen, B. , & Finocchiaro, R. (2016). Temperature and hydrology affect methane emissions from Prairie Pothole Wetlands. Wetlands, 36(2), 371–381. 10.1007/s13157-016-0826-8

[gcb16137-bib-0007] Bengtson, P. , Sterngren, A. E. , & Rousk, J. (2012). Archaeal abundance across a PH gradient in an arable soil and its relationship to bacterial and fungal growth rates. Applied and Environmental Microbiology, 78(16), 5906–5911. 10.1128/AEM.01476-12 22706045 PMC3406126

[gcb16137-bib-0008] Beylich, A. (2003). Present morphoclimates and morphodynamics in Latnjavagge, the Northern Swedish Lapland and Sustdalur, East Iceland. Jökull, 52(1), 33–54.

[gcb16137-bib-0009] Beylich, A. A. , Kolstrup, E. , Thyrsted, T. , Linde, N. , Pedersen, L. B. , & Dynesius, L. (2004). Chemical denudation in arctic‐alpine Latnjavagge (Swedish Lapland) in relation to regolith as assessed by radio magnetotelluric‐geophysical profiles s. Geomorphology, 57(3–4), 303–319. 10.1016/S0169-555X(03)00162-4

[gcb16137-bib-0010] Biskaborn, B. K. , Smith, S. L. , Noetzli, J. , Matthes, H. , Vieira, G. , Streletskiy, D. A. , Schoeneich, P. , Romanovsky, V. E. , Lewkowicz, A. G. , Abramov, A. , Allard, M. , Boike, J. , Cable, W. L. , Christiansen, H. H. , Delaloye, R. , Diekmann, B. , Drozdov, D. , Etzelmüller, B. , Grosse, G. , … Lantuit, H. (2019). Permafrost is warming at a global scale. Nature Communications, 10(1), 1–11. 10.1038/s41467-018-08240-4 PMC6335433 30651568

[gcb16137-bib-0011] Björk, R. G. , Majdi, H. , Klemedtsson, L. , Lewis‐Jonsson, L. , & Molau, U. (2007). Long‐term warming effects on root morphology, root mass distribution, and microbial activity in two dry tundra plant communities in Northern Sweden. New Phytologist, 176(4), 862–873. 10.1111/j.1469-8137.2007.02231.x 17937761

[gcb16137-bib-0012] Bjorkman, A. D. , Myers‐Smith, I. H. , Elmendorf, S. C. , Normand, S. , Rüger, N. , Beck, P. S. A. , Blach‐Overgaard, A. , Blok, D. , Cornelissen, J. H. C. , Forbes, B. C. , Georges, D. , Goetz, S. J. , Guay, K. C. , Henry, G. H. R. , HilleRisLambers, J. , Hollister, R. D. , Karger, D. N. , Kattge, J. , Manning, P. , … Weiher, E. (2018). Plant functional trait change across a warming tundra biome. Nature, 562(7725), 57–62. 10.1038/s41586-018-0563-7 30258229

[gcb16137-bib-0013] Blanc‐Betes, E. , Welker, J. M. , Sturchio, N. C. , Chanton, J. P. , & Gonzalez‐Meler, M. A. (2016). Winter precipitation and snow accumulation drive the methane sink or source strength of Arctic tussock tundra. Global Change Biology, 22(8), 2818–2833. 10.1111/gcb.13242 26851545

[gcb16137-bib-0014] Blois, J. L. , Williams, J. W. , Fitzpatrick, M. C. , Jackson, S. T. , & Ferrier, S. (2013). space can substitute for time in predicting climate‐change effects on biodiversity. Proceedings of the National Academy of Sciences of the United States of America, 110(23), 9374–9379. 10.1073/pnas.1220228110 23690569 PMC3677423

[gcb16137-bib-0015] Boyd, J. A. , Woodcroft, B. J. , & Tyson, G. W. (2018). GraftM: A tool for scalable, phylogenetically informed classification of genes within metagenomes. Nucleic Acids Research, 46(10), e59. 10.1093/nar/gky174 29562347 PMC6007438

[gcb16137-bib-0016] Bring, A. , Fedorova, I. , Dibike, Y. , Hinzman, L. , Mård, J. , Mernild, S. H. , Prowse, T. , Semenova, O. , Stuefer, S. L. , & Woo, M. K. (2016). Arctic terrestrial hydrology: A synthesis of processes, regional effects, and research challenges. Journal of Geophysical Research G: Biogeosciences, 121(3), 621–649. 10.1002/2015JG003131

[gcb16137-bib-0017] Brix, H. , Sorrell, B. K. , & Lorenzen, B. (2001). Are phragmites‐dominated wetlands a net source or net sink of greenhouse gases? Aquatic Botany, 69(2–4), 313–324. 10.1016/S0304-3770(01)00145-0

[gcb16137-bib-0018] Callaghan, T. V. , Bergholm, F. , Christensen, T. R. , Jonasson, C. , Kokfelt, U. , & Johansson, M. (2010). A new climate era in the sub‐Arctic: Accelerating climate changes and multiple impacts. Geophysical Research Letters, 37(14), 1–6. 10.1029/2009GL042064

[gcb16137-bib-0019] Chapin, F. S. , Van Cleve, K. , & Chapin, M. C. (1979). Soil temperature and nutrient cycling in the tussock growth form of *Eriophorum vaginatum* . Journal of Ecology, 67, 169–189.– 10.2307/2259343

[gcb16137-bib-0020] Christensen, T. R. , Friborg, T. , Sommerkorn, M. , Kaplan, J. , Illeris, L. , Soegaard, H. , Nordstroem, C. , & Jonasson, S. (2000). Trace gas exchange in a high‐Arctic valley: 1. Variationsin CO_2_ and CH_4_ flux between tundra vegetation types. Global Biogeochemical Cycles, 14(3), 701–713. 10.1029/1999GB001134

[gcb16137-bib-0021] Coolen, M. J. L. , & Orsi, W. D. (2015). The transcriptional response of microbial communities in thawing Alaskan permafrost soils. Frontiers in Microbiology, 6. 10.3389/fmicb.2015.0019 PMC4360760 25852660

[gcb16137-bib-0022] Corradi, C. , Kolle, O. , Walter, K. , Zimov, S. A. , & Schulze, E. D. (2005). Carbon dioxide and methane exchange of a North‐East Siberian tussock tundra. Global Change Biology, 11(11), 1910–1925. 10.1111/j.1365-2486.2005.01023.x

[gcb16137-bib-0023] D’Imperio, L. , Nielsen, C. S. , Westergaard‐Nielsen, A. , Michelsen, A. , & Elberling, B. O. (2017). Methane oxidation in contrasting soil types: Responses to experimental warming with implication for landscape‐integrated CH_4_ budget. Global Change Biology, 23(2), 966–976. 10.1111/gcb.13400 27416869

[gcb16137-bib-0024] Dalal, R. C. , Allen, D. E. , Livesley, S. J. , & Richards, G. (2008). Magnitude and biophysical regulators of methane emission and consumption in the Australian agricultural, forest, and submerged landscapes: A review. Plant and Soil, 309(1–2), 43–76. 10.1007/s11104-007-9446-7

[gcb16137-bib-0025] Davidson, E. A. , & Janssens, I. A. (2006). Temperature sensitivity of soil carbon decomposition and feedbacks to climate change. Nature, 440(7081), 165–173. 10.1038/nature04514 16525463

[gcb16137-bib-0026] Edgar, R. C. , Haas, B. J. , Clemente, J. C. , Quince, C. , & Knight, R. (2011). UCHIME improves sensitivity and speed of chimera detection. Bioinformatics, 27(16), 2194–2200. 10.1093/bioinformatics/btr381 21700674 PMC3150044

[gcb16137-bib-0027] Elberling, B. O. , Michelsen, A. , Schädel, C. , Schuur, E. A. G. , Christiansen, H. H. , Berg, L. , Tamstorf, M. P. , & Sigsgaard, C. (2013). Long‐term CO_2_ production following permafrost thaw. Nature Climate Change, 3(10), 890–894. 10.1038/nclimate1955

[gcb16137-bib-0028] Emmerton, C. A. , St, V. L. , Louis, I. , Lehnherr, E. R. , Humphreys, E. R. , & Kosolofski, H. R. (2014). The net exchange of methane with high Arctic landscapes during the summer growing season. Biogeosciences, 11(12), 3095–3106. 10.5194/bg-11-3095-2014

[gcb16137-bib-0029] Eren, A. M. , Vineis, J. H. , Morrison, H. G. , & Sogin, M. L. (2013). A filtering method to generate high quality short reads using illumina paired‐end technology. PLoS One, 8(6), 6–11. 10.1371/journal.pone.0066643 PMC3684618 23799126

[gcb16137-bib-0030] Etzelmüller, B. , Flo, E. S. , Heggem, N. S. , Frauenfelder, R. , Kääb, A. , & Goulden, C. (2006). Mountain Permafrost distribution modelling using a multi‐criteria approach in the Hövsgöl area, Northern Mongolia. Permafrost and Periglacial Processes, 17(2), 91–104. 10.1002/ppp.554

[gcb16137-bib-0031] Fan, Y. , Miguez‐Macho, G. , Jobbágy, E. G. , Jackson, R. B. , & Otero‐Casal, C. (2017). Hydrologic regulation of plant rooting depth. Proceedings of the National Academy of Sciences of the United States of America, 114(40), 10572–10577. 10.1073/pnas.1712381114 28923923 PMC5635924

[gcb16137-bib-0032] Gebauer, R. L. , Tenhunen, J. D. , & Reynolds, J. F. (1996). Soil aeration in relation to soil physical properties, nitrogen availability, and root characteristics within an Arctic watershed. Plant and Soil, 178(1), 37–48. 10.1007/BF00011161

[gcb16137-bib-0033] Gisnås, K. , Etzelmüller, B. , Lussana, C. , Hjort, J. , Sannel, A. B. K. , Isaksen, K. , Westermann, S. , Kuhry, P. , Christiansen, H. H. , Frampton, A. , & Åkerman, J. (2017). Permafrost map for Norway, Sweden and Finland. Permafrost and Periglacial Processes, 28(2), 359–378. 10.1002/ppp.1922

[gcb16137-bib-0034] Greenup, A. L. , Bradford, M. A. , Mcnamara, N. P. , Ineson, P. , & Lee, J. A. (2000). The role of *Eriophorum vaginatum* in CH_4_ flux from an ombrotrophic peatland. Plant and Soil, 227(1–2), 265–272. 10.1023/A:1026573727311

[gcb16137-bib-0035] Griffiths, R. I. , Whiteley, A. S. , O’Donnell, A. G. , & Bailey, M. J. (2000). Rapid method for coextraction of DNA and RNA from natural environments for analysis of ribosomal DNA‐ and RRNA‐based microbial community composition. Applied and Environmental Microbiology, 66(12), 5488–5491. 10.1128/AEM.66.12.5488-5491.2000 11097934 PMC92488

[gcb16137-bib-0036] Grogan, P. , & Jonasson, S. (2005). Temperature and substrate controls on intra‐annual variation in ecosystem respiration in two subarctic vegetation type. Global Change Biology, 11(3), 465–475. 10.1111/j.1365-2486.2005.00912.x

[gcb16137-bib-0037] Haeberli, W. , Noetzli, J. , Arenson, L. , Delaloye, R. , Gärtner‐Roer, I. , Gruber, S. , Isaksen, K. , Kneisel, C. , Krautblatter, M. , & Phillips, M. (2011). Mountain permafrost: Development and challenges of a young research field. Journal of Glaciology, 56(200), 1043–1058. 10.3189/002214311796406121

[gcb16137-bib-0038] Henneberg, A. , Sorrell, B. K. , & Brix, H. (2012). Internal methane transport through *Juncus effusus*: experimental manipulation of morphological barriers to test above‐ and below‐ground diffusion limitation. New Phytologist, 196(3), 799–806. 10.1111/j.1469-8137.2012.04303.x 22966782

[gcb16137-bib-0039] Henry, G. H. R. , & Molau, U. (1997). Tundra plants and climate change: the International Tundra Experiment (ITEX). Global Change Biology, 3(SUPPL. 1), 1–9. 10.1111/j.1365-2486.1997.gcb132.x

[gcb16137-bib-0040] Holmes, A. J. , Roslev, P. , McDonald, I. R. , Iversen, N. , Henriksen, K. , & Colin Murrell, J. (1999). Characterization of methanotrophic bacterial populations in soils showing atmospheric methane uptake. Applied and Environmental Microbiology, 65(8), 3312–3318. 10.1128/aem.65.8.3312-3318.1999 10427012 PMC91497

[gcb16137-bib-0041] Hugelius, G. , Strauss, J. , Zubrzycki, S. , Harden, J. W. , Schuur, E. A. G. , Ping, C.‐L. , Schirrmeister, L. , Grosse, G. , Michaelson, G. J. , Koven, C. D. , O'Donnell, J. A. , Elberling, B. , Mishra, U. , Camill, P. , Yu, Z. , Palmtag, J. , & Kuhry, P. (2014). Estimated stocks of circumpolar permafrost carbon with quantified uncertainty ranges and identified data gaps. Biogeosciences, 11(23), 6573–6593. 10.5194/bg-11-6573-2014

[gcb16137-bib-0042] van Huissteden, J. , Berrittella, C. , Parmentier, F. J. W. , Mi, Y. , Maximov, T. C. , & Dolman, A. J. (2011). Methane emissions from permafrost thaw lakes limited by lake drainage. Nature Climate Change, 1(2), 119–123. 10.1038/nclimate1101

[gcb16137-bib-0043] Iversen, C. M. , Sloan, V. L. , Sullivan, P. F. , Euskirchen, E. S. , David Mcguire, A. , Norby, R. J. , Walker, A. P. , Warren, J. M. , & Wullschleger, S. D. (2015). The unseen iceberg: plant roots in arctic tundra. New Phytologist, 205(1), 34–58. 10.1111/nph.13003 25209220

[gcb16137-bib-0044] Johansson, M. , Åkerman, J. , Keuper, F. , Christensen, T. R. , Lantuit, H. , & Callaghan, T. V. (2011). Past and present permafrost temperatures in the Abisko area: Redrilling of boreholes. Ambio, 40(6), 558–565. 10.1007/s13280-011-0163-3 21954719 PMC3357866

[gcb16137-bib-0045] Johansson, M. , Christensen, T. R. , Jonas Akerman, H. , & Callaghan, T. V. (2006). What determines the current presence or absence of permafrost in the Torneträsk Region, a sub‐arctic landscape in Northern Sweden? Ambio, 35(4), 190–197. 16944644 10.1579/0044-7447(2006)35[190:wdtcpo]2.0.co;2

[gcb16137-bib-0046] Jørgensen, J. , Christian, K. M. , Johansen, L. , Westergaard‐Nielsen, A. , & Elberling, B. O. (2015). Net regional methane sink in high Arctic soils of Northeast Greenland. Nature Geoscience, 8(1), 20–23. 10.1038/ngeo2305

[gcb16137-bib-0047] Katoh, K. , Misawa, K. , Kuma, K. I. , & Miyata, T. (2002). MAFFT: A Novel method for rapid multiple sequence alignment based on fast Fourier transform. Nucleic Acids Research, 30(14), 3059–3066. 10.1093/nar/gkf436 12136088 PMC135756

[gcb16137-bib-0048] Kolb, S. (2009). The quest for atmospheric methane oxidizers in forest soils. Environmental Microbiology Reports, 1(5), 336–346. 10.1111/j.1758-2229.2009.00047.x 23765885

[gcb16137-bib-0049] Kwon, M. J. , Beulig, F. , Ilie, I. , Wildner, M. , Küsel, K. , Merbold, L. , Mahecha, M. D. , Zimov, N. , Zimov, S. A. , Heimann, M. , Schuur, E. A. G. , Kostka, J. E. , Kolle, O. , Hilke, I. , & Göckede, M. (2017). Plants, microorganisms, and soil temperatures contribute to a decrease in methane fluxes on a drained arctic floodplain. Global Change Biology, 23(6), 2396–2412. 10.1111/gcb.13558 27901306

[gcb16137-bib-0050] Kwon, M. J. , Heimann, M. , Kolle, O. , Luus, K. A. , Schuur, E. A. G. , Zimov, N. , Zimov, S. A. , & Göckede, M. (2016). Long‐term drainage reduces CO_2_ uptake and increases CO_2_ emission on a Siberian floodplain due to shifts in vegetation community and soil thermal characteristics. Biogeosciences, 13(14), 4219–4235. 10.5194/bg-13-4219-2016

[gcb16137-bib-0051] Kwon, M. J. , Jung, J. Y. , Tripathi, B. M. , Göckede, M. , Lee, Y. K. , & Kim, M. (2019). Dynamics of microbial communities and CO_2_ and CH_4_ fluxes in the tundra ecosystems of the changing Arctic. Journal of Microbiology, 57(5), 325–336. 10.1007/s12275-019-8661-2 30656588

[gcb16137-bib-0052] Kwon, M. J. , Tripathi, B. M. , Göckede, M. , Shin, S. C. , Myeong, N. R. , Lee, Y. K. , & Kim, M. (2021). Disproportionate microbial responses to decadal drainage on a Siberian floodplain. Global Change Biology, 27(20), 5124–5140. 10.1111/gcb.15785 34216067

[gcb16137-bib-0053] Lau, M. C. Y. , Stackhouse, B. T. , Layton, A. C. , Chauhan, A. , Vishnivetskaya, T. A. , Chourey, K. , Ronholm, J. , Mykytczuk, N. C. S. , Bennett, P. C. , Lamarche‐Gagnon, G. , Burton, N. , Pollard, W. H. , Omelon, C. R. , Medvigy, D. M. , Hettich, R. L. , Pfiffner, S. M. , Whyte, L. G. , & Onstott, T. C. (2015). An active atmospheric methane sink in high arctic mineral cryosols. The ISME Journal, 9(8), 1880–1891. 10.1038/ismej.2015.13 25871932 PMC4511939

[gcb16137-bib-0054] Lawrence, D. M. , Koven, C. D. , Swenson, S. C. , Riley, W. J. , & Slater, A. G. (2015). Permafrost thaw and resulting soil moisture changes regulate projected high‐latitude CO_2_ and CH_4_ emissions. Environmental Research Letters, 10(9), 10.1088/1748-9326/10/9/094011. 094011.

[gcb16137-bib-0055] Le Mer, J. , & Roger, P. (2001). Production, oxidation, emission and consumption of methane by soils: A review. European Journal of Soil Biology, 37(1), 25–50. 10.1016/S1164-5563(01)01067-6

[gcb16137-bib-0056] Lupascu, M. , Wadham, J. , Hornibrook, E. , & Pancost, R. (2012). Temperature sensitivity of methane production in the permafrost active layer at Stordalen, Sweden: A comparison with non‐permafrost northern wetlands. Arctic, Antarctic, and Alpine Research, 44(4), 469–482. 10.1657/1938-4246-44.4.469

[gcb16137-bib-0057] Luton, P. E. , Wayne, J. M. , Sharp, R. J. , & Riley, P. W. (2002). The McrA gene as an alternative to 16S RRNA in the phylogenetic analysis of methanogen populations in landfill. Microbiology, 148(11), 3521–3530. 10.1099/00221287-148-11-3521 12427943

[gcb16137-bib-0058] Margesin, R. , & Collins, T. (2019). Microbial ecology of the cryosphere (glacial and permafrost habitats): Current knowledge. Applied Microbiology and Biotechnology, 103(6), 2537–2549. 10.1007/s00253-019-09631-3 30719551 PMC6443599

[gcb16137-bib-0059] Martin, A. C. , Jeffers, E. S. , Petrokofsky, G. , Myers‐Smith, I. , & MacIas‐Fauria, M. (2017). Shrub growth and expansion in the Arctic tundra: An assessment of controlling factors using an evidence‐based approach. Environmental Research Letters, 12(8), 10.1088/1748-9326/aa7989

[gcb16137-bib-0060] Mastepanov, M. , Sigsgaard, C. , Dlugokencky, E. J. , Houweling, S. , Ström, L. , Tamstorf, M. P. , & Christensen, T. R. (2008). Large tundra methane burst during onset of freezing. Nature, 456(7222), 628–630. 10.1038/nature07464 19052625

[gcb16137-bib-0061] Matveev, A. , Laurion, I. , & Vincent, W. F. (2018). Methane and carbon dioxide emissions from Thermokarst lakes on mineral soils. Arctic Science, 4(4), 584–604. 10.1139/as-2017-0047

[gcb16137-bib-0062] McDonald, I. R. , Bodrossy, L. , Chen, Y. , & Colin Murrell, J. (2008). Molecular ecology techniques for the study of aerobic methanotrophs. Applied and Environmental Microbiology, 74(5), 1305–1315. 10.1128/AEM.02233-07 18165358 PMC2258629

[gcb16137-bib-0063] McMurdie, P. J. , & Holmes, S. (2013). Phyloseq: An R package for reproducible interactive analysis and graphics of microbiome census data. PLoS One, 8(4), e61217.– 10.1371/journal.pone.0061217 23630581 PMC3632530

[gcb16137-bib-0064] Mekonnen, Z. A. , Riley, W. J. , & Grant, R. F. (2018). 21st century tundra shrubification could enhance net carbon uptake of North America Arctic tundra under an RCP8.5 climate trajectory. Environmental Research Letters, 13(5), 10.1088/1748-9326/AABF28. 054029.

[gcb16137-bib-0065] Merbold, L. , Kutsch, W. L. , Corradi, C. , Kolle, O. , Rebmann, C. , Stoy, P. C. , Zimov, S. A. , & Schulze, E. D. (2009). artificial drainage and associated carbon fluxes (CO_2_/CH_4_) in a tundra ecosystem. Global Change Biology, 15(11), 2599–2614. 10.1111/j.1365-2486.2009.01962.x

[gcb16137-bib-0066] Messan, K. S. , Jones, R. M. , Doherty, S. J. , Foley, K. , Douglas, T. A. , & Barbato, R. A. (2020). The role of changing temperature in microbial metabolic processes during permafrost thaw. PLoS One, 15(4), 1–20. 10.1371/journal.pone.0232169 PMC7192436 32353013

[gcb16137-bib-0067] Moguel, G. , Regina, A. M. , Bass, M. H. , Garnett, M. P. , Keenan, B. , Matveev, A. , & Douglas, P. M. J. (2021). Radiocarbon data reveal contrasting sources for carbon fractions in thermokarst lakes and rivers of Eastern Canada (Nunavik, Quebec). Journal of Geophysical Research: Biogeosciences, 126(4). 10.1029/2020JG005938

[gcb16137-bib-0068] Molau, U. (2010). Long‐term impacts of observed and induced climate change on tussock tundra near its southern limit in Northern Sweden. Plant Ecology and Diversity, 3(1), 29–34. 10.1080/17550874.2010.487548

[gcb16137-bib-0069] Molau, U. , & Mølgaard, P. (1996). International tundra experiment ITEX manual (2nd edn.). Danish Polar Center.

[gcb16137-bib-0070] Natali, S. M. , Holdren, J. P. , Rogers, B. M. , Treharne, R. , Duffy, P. B. , Pomerance, R. , & MacDonald, E. (2021). Permafrost carbon feedbacks threaten global climate goals. Proceedings of the National Academy of Sciences of the United States of America, 118(21), 1–3. 10.1073/pnas.2100163118 PMC8166174 34001617

[gcb16137-bib-0071] Nazaries, L. , Colin Murrell, J. , Millard, P. , Baggs, L. , & Singh, B. K. (2013). Methane, microbes and models: Fundamental understanding of the soil methane cycle for future predictions. Environmental Microbiology, 15(9), 2395–2417. 10.1111/1462-2920.12149 23718889

[gcb16137-bib-0072] Nielsen, C. S. , Michelsen, A. , Per Ambus, T. K. K. , Deepagoda, C. , & Elberling, B. O. (2017). Linking rhizospheric CH_4_ oxidation and net CH_4_ emissions in an Arctic wetland based on ^13^CH_4_ labeling of mesocosms. Plant and Soil, 412(1–2), 201–213. 10.1007/s11104-016-3061-4

[gcb16137-bib-0073] Oberbauer, S. F. , Starr, G. , & Pop, E. W. (1998). Effects of extended growing season and soil warming on carbon dioxide and methane exchange of Tussock tundra in Alaska. Journal of Geophysical Research Atmospheres, 103(D22), 29075–29082. 10.1029/98JD00522

[gcb16137-bib-0074] Ødegård, R. S. , Hoelzle, M. , Johansen, K. V. , & Sollid, J. L. (1996). Permafrost mapping and prospecting in southern Norway. Norsk Geografisk Tidsskrift, 50(1), 41–53. 10.1080/00291959608552351

[gcb16137-bib-0075] Oh, Y. , Stackhouse, B. , Lau, M. C. Y. , Xu, X. , Trugman, A. T. , Moch, J. , Onstott, T. C. , Jørgensen, C. J. , D'Imperio, L. , Elberling, B. O. , Emmerton, C. A. , St. Louis, V. L. , & Medvigy, D. (2016). A scalable model for methane consumption in Arctic mineral soils. Geophysical Research Letters, 43(10), 5143–5150. 10.1002/2016GL069049

[gcb16137-bib-0076] Oh, Y. , Zhuang, Q. , Liu, L. , Welp, L. R. , Lau, M. C. Y. , Onstott, T. C. , Medvigy, D. , Bruhwiler, L. , Dlugokencky, E. J. , Hugelius, G. , D’Imperio, L. , & Elberling, B. O. (2020). Reduced net methane emissions due to microbial methane oxidation in a warmer Arctic. Nature Climate Change, 10(4), 317–321. 10.1038/s41558-020-0734-z

[gcb16137-bib-0077] Olefeldt, D. , Turetsky, M. R. , Crill, P. M. , & David Mcguire, A. (2013). Environmental and physical controls on northern terrestrial methane emissions across permafrost zones. Global Change Biology, 19(2), 589–603. 10.1111/gcb.12071 23504795

[gcb16137-bib-0078] Pérez, C. A. , DeGrandpre, M. D. , Lagos, N. A. , Saldías, G. S. , Cascales, E.‐K. , & Vargas, C. A. (2015). Effects of simulated spring thaw of permafrost from mineral cryosol on CO_2_ emissions and atmospheric CH_4_ uptake. Journal of Geophysical Research: Biogeosciences, 120(Iii), 673–692. 10.1002/2015JG003004.Received

[gcb16137-bib-0079] R Core Team . (2019). A language and environment for statistical computing. R Foundation for Statistical Computing.

[gcb16137-bib-0080] Ridefelt, H. , Etzelmüller, B. , Boelhouwers, J. , & Jonasson, C. (2008). Statistic‐empirical modelling of mountain permafrost distribution in the Abisko region, Sub‐Arctic Northern Sweden. Norsk Geografisk Tidsskrift, 62(4), 278–289. 10.1080/00291950802517890

[gcb16137-bib-0081] Sachs, T. , Wille, C. , Boike, J. , & Kutzbach, L. (2008). Environmental controls on ecosystem‐scale CH_4_ emission from polygonal tundra in the Lena River Delta, Siberia. Journal of Geophysical Research: Biogeosciences, 113(3), 1–12. 10.1029/2007JG000505

[gcb16137-bib-0082] Scharn, R. , Brachmann, C. G. , Patchett, A. , Reese, H. , Bjorkman, A. , Alatalo, J. , Björk, R. G. , Jägerbrand, A. K. , Molau, U. , & Björkman, M. P. (2021). Vegetation responses to 26 years of warming at Latnjajaure Field Station, Northern Sweden. Arctic Science, 20(April), 1–20. 10.1139/as-2020-0042

[gcb16137-bib-0083] Scharn, R. , Little, C. J. , Bacon, C. D. , Alatalo, J. M. , Antonelli, A. , Björkman, M. P. , Ulf Molau, R. , Nilsson, H. , & Björk, R. G. (2021). Decreased soil moisture due to warming drives phylogenetic diversity and community transitions in the tundra. Environmental Research Letters, 16(6), 10.1088/1748-9326/abfe8a

[gcb16137-bib-0084] Schuur, E. A. G. , Bracho, R. , Celis, G. , Belshe, E. F. , Ebert, C. , Ledman, J. , Mauritz, M. , Pegoraro, E. F. , Plaza, C. , Rodenhizer, H. , Romanovsky, V. , Schädel, C. , Schirokauer, D. , Taylor, M. , Vogel, J. G. , & Webb, E. E. (2021). Tundra underlain by thawing permafrost persistently emits carbon to the atmosphere over 15 years of measurements. Journal of Geophysical Research: Biogeosciences, 126(6), e2020JG006044. 10.1029/2020JG006044

[gcb16137-bib-0085] Schuur, E. A. G. , McGuire, A. D. , Schädel, C. , Grosse, G. , Harden, J. W. , Hayes, D. J. , Hugelius, G. , Koven, C. D. , Kuhry, P. , Lawrence, D. M. , Natali, S. M. , Olefeldt, D. , Romanovsky, V. E. , Schaefer, K. , Turetsky, M. R. , Treat, C. C. , & Vonk, J. E. (2015). Climate change and the permafrost carbon feedback. Nature, 520(7546), 171–179. 10.1038/nature14338 25855454

[gcb16137-bib-0086] Schuur, E. A. G. , Vogel, J. G. , Crummer, K. G. , Lee, H. , Sickman, J. O. , & Osterkamp, T. E. (2009). The effect of permafrost thaw on old carbon release and net carbon exchange from tundra. Nature, 459(7246), 556–559. 10.1038/nature08031 19478781

[gcb16137-bib-0087] Segers, R. (1998). Methane production and methane consumption: a review of processes underlying wetland fluxes. Biogeochemistry, 41, 23–51.

[gcb16137-bib-0088] Serrano‐Silva, N. , Sarria‐Guzmán, Y. , Dendooven, L. , & Luna‐Guido, M. (2014). Methanogenesis and methanotrophy in soil: A review. Pedosphere, 24(3), 291–307. 10.1016/S1002-0160(14)60016-3

[gcb16137-bib-0089] Stackhouse, B. , Lau, M. C. Y. , Vishnivetskaya, T. , Burton, N. , Wang, R. , Southworth, A. , Whyte, L. , & Onstott, T. C. (2017). Atmospheric CH_4_ oxidation by Arctic permafrost and mineral cryosols as a function of water saturation and temperature. Geobiology, 15(1), 94–111. 10.1111/gbi.12193 27474434

[gcb16137-bib-0090] Ström, L. , Ekberg, A. , Mastepanov, M. , & Christensen, T. R. (2003). The effect of vascular plants on carbon turnover and methane emissions from a tundra wetland. Global Change Biology, 9, 1185–1192. 10.1046/j.1365-2486.2003.00655.x

[gcb16137-bib-0091] Sturtevant, C. S. , Oechel, W. C. , Zona, D. , Kim, Y. , & Emerson, C. E. (2012). Soil moisture control over autumn season methane flux, Arctic Coastal Plain of Alaska. Biogeosciences, 9(4), 1423–1440. 10.5194/bg-9-1423-2012

[gcb16137-bib-0092] Tarnocai, C. , Canadell, J. G. , Schuur, E. A. G. , Kuhry, P. , Mazhitova, G. , & Zimov, S. (2009). Soil organic carbon pools in the northern circumpolar permafrost region. Global Biogeochemical Cycles, 23(2), 1–11. 10.1029/2008GB003327

[gcb16137-bib-0093] Torn, M. S. , & Stuart Chapin, F. (1993). Environmental and biotic controls over methane flux from Arctic tundra. Chemosphere, 26(1–4), 357–368. 10.1016/0045-6535(93)90431-4

[gcb16137-bib-0094] Wagner, D. , Kobabe, S. , Pfeiffer, E. M. , & Hubberten, H. W. (2003). Microbial controls on methane fluxes from a polygonal tundra of the Lena Delta, Siberia. Permafrost and Periglacial Processes, 14(2), 173–185. 10.1002/ppp.443

[gcb16137-bib-0095] Wagner, D. , Lipski, A. , Embacher, A. , & Gattinger, A. (2005). Methane fluxes in permafrost habitats of the Lena Delta: Effects of microbial community structure and organic matter quality. Environmental Microbiology, 7(10), 1582–1592. 10.1111/j.1462-2920.2005.00849.x 16156731

[gcb16137-bib-0096] Walker, D. A. , Raynolds, M. K. , Daniëls, F. J. A. , Einarsson, E. , Elvebakk, A. , Gould, W. A. , Katenin, A. E. , Kholod, S. S. , Markon, C. J. , Melnikov, E. S. , Moskalenko, N. G. , Talbot, S. S. , Yurtsev, B. A(†). , & The other members of The CAVM Team . (2005). The circumpolar arctic vegetation map. Journal of Vegetation Science, 16(3), 267–282. 10.1111/j.1654-1103.2005.tb02365.x

[gcb16137-bib-0097] Walker, M. D. , Henrik Wahren, C. , Hollister, R. D. , Henry, G. H. R. , Ahlquist, L. E. , Alatalo, J. M. , Syndonia Bret‐Harte, M. et al (2006). Plant community responses to experimental warming across the tundra biome. Proceedings of the National Academy of Sciences of the United States of America, 103(5), 1342–1346. 10.1073/pnas.0503198103 16428292 PMC1360515

[gcb16137-bib-0098] Walters, W. , Hyde, E. R. , Berg‐Lyons, D. , Ackermann, G. , Humphrey, G. , Parada, A. , Gilbert, J. A. , Jansson, J. K. , Caporaso, J. G. , Fuhrman, J. A. , Apprill, A. , & Knight, R. (2015). Improved bacterial 16S RRNA gene (V4 and V4–5) and fungal internal transcribed spacer marker gene primers for microbial community surveys. Msystems, 1(1), e0009–15. 10.1128/mSystems.00009-15.Editor PMC5069754 27822518

[gcb16137-bib-0099] Wang, Q. , Garrity, G. M. , Tiedje, J. M. , & Cole, J. R. (2007). Naïve Bayesian classifier for rapid assignment of RRNA sequences into the new bacterial taxonomy. Applied and Environmental Microbiology, 73(16), 5261–5267. 10.1128/AEM.00062-07 17586664 PMC1950982

[gcb16137-bib-0100] Watanabe, K. , Kodama, Y. , & Harayama, S. (2001). Design and evaluation of PCR primers to amplify bacterial 16S ribosomal DNA fragments used for community fingerprinting. Journal of Microbiological Methods, 44(3), 253–262. 10.1016/S0167-7012(01)00220-2 11240048

[gcb16137-bib-0101] Watanabe, T. , Kimura, M. , & Asakawa, S. (2007). Dynamics of methanogenic archaeal communities based on RRNA analysis and their relation to methanogenic activity in Japanese paddy field soils. Soil Biology and Biochemistry, 39(11), 2877–2887. 10.1016/j.soilbio.2007.05.030

[gcb16137-bib-0102] Wei, S. , Cui, H. , Zhu, Y. , Zhenquan, L. U. , Pang, S. , Zhang, S. , Dong, H. , & Xin, S. U. (2018). Shifts of methanogenic communities in response to permafrost thaw results in rising methane emissions and soil property changes. Extremophiles, 22(3), 447–459. 10.1007/s00792-018-1007-x 29429010

[gcb16137-bib-0103] Westermann, S. , Elberling, B. , Højlund Pedersen, S. , Stendel, M. , Hansen, B. U. , & Liston, G. E. (2015). Future permafrost conditions along environmental gradients in Zackenberg, Greenland. Cryosphere, 9(2), 719–735. 10.5194/tc-9-719-2015

[gcb16137-bib-0104] Whalen, S. C. , & Reeburgh, W. S. (1990). Comsumption of atmospheric methane by tundra soils. Nature, 346, 160–162.

[gcb16137-bib-0105] Wilson, G. V. , Thiesse, B. R. , & Scott, H. D. (1985). Relationships among oxygen flux, soil water tension, and aeration porosity in a drying soil profile. Soil Science, 139(1).

[gcb16137-bib-0106] Yang, S. , Wen, X. , & Liebner, S. (2016). PmoA gene reference database (fasta‐formatted sequences and taxonomy). 10.5880/GFZ.5.3.2016.001

[gcb16137-bib-0107] Yang, Z. , Hanna, E. , Callaghan, T. V. , & Jonasson, C. (2012). How Can meteorological observations and microclimate simulations improve understanding of 1913–2010 climate change around Abisko, Swedish Lapland? Meteorological Applications, 19(4), 454–463. 10.1002/met.276

[gcb16137-bib-0108] Yang, Z. , Yang, S. , Van Nostrand, J. D. , Zhou, J. , Fang, W. , Qi, Q. , Liu, Y. , Wullschleger, S. D. , Liang, L. , Graham, D. E. , Yang, Y. , & Gu, B. (2017). Microbial community and functional gene changes in Arctic tundra soils in a microcosm warming experiment. Frontiers in Microbiology, 8(SEP), 1–11. 10.3389/fmicb.2017.01741 28974946 PMC5610689

[gcb16137-bib-0109] Zona, D. (2016). Long‐term effects of permafrost thaw. Nature, 537(7622), 625–626. 10.1038/537625a 27680935

[gcb16137-bib-0110] Zona, D. , Gioli, B. , Commane, R. , Lindaas, J. , Wofsy, S. C. , Miller, C. E. , Dinardo, S. J. , Dengel, S. , Sweeney, C. , Karion, A. , Chang, R.‐W. , Henderson, J. M. , Murphy, P. C. , Goodrich, J. P. , Moreaux, V. , Liljedahl, A. , Watts, J. D. , Kimball, J. S. , Lipson, D. A. , & Oechel, W. C. (2016). Cold season emissions dominate the arctic tundra methane budget. Proceedings of the National Academy of Sciences of the United States of America, 113(1), 40–45. 10.1073/pnas.1516017113 26699476 PMC4711884

[gcb16137-bib-0111] Zona, D. , Oechel, W. C. , Kochendorfer, J. , Paw U, K. T. , Salyuk, A. N. , Olivas, P. C. , Oberbauer, S. F. , & Lipson, D. A. (2009). Methane fluxes during the initiation of a large‐scale water table manipulation experiment in the Alaskan arctic tundra. Global Biogeochemical Cycles, 23(2), 1–11. 10.1029/2009GB003487

