# Supplementary material for: Reduced methane emissions in former permafrost soils driven by vegetation and microbial changes following drainage
Source: Glob Chang Biol. 2022 Mar 14;28(10):3411–25. doi: 10.1111/gcb.16137 (PMC9314937; doi:10.1111/gcb.16137)
Supplement: Supplementary file 1 — Supplementary Material [file GCB-28-3411-s001.docx]

**Supplementary Information for**

Reduced methane emissions in former permafrost soils driven by vegetation and microbial changes following drainage

Christoph Keuschnig, Catherine Larose, Mario Rudner, Argus Pesqueda, Stéphane Doleac, Bo Elberling, Robert G. Björk, Leif Klemedtsson and Mats P. Björkman

Corresponding author: Mats P. Björkman

Email: [mats.bjorkman@gu.se](mailto:mats.bjorkman@gu.se)

**This PDF file includes:**

Figures S1 to S3

Tables S1 to S3

SI References: 1-27


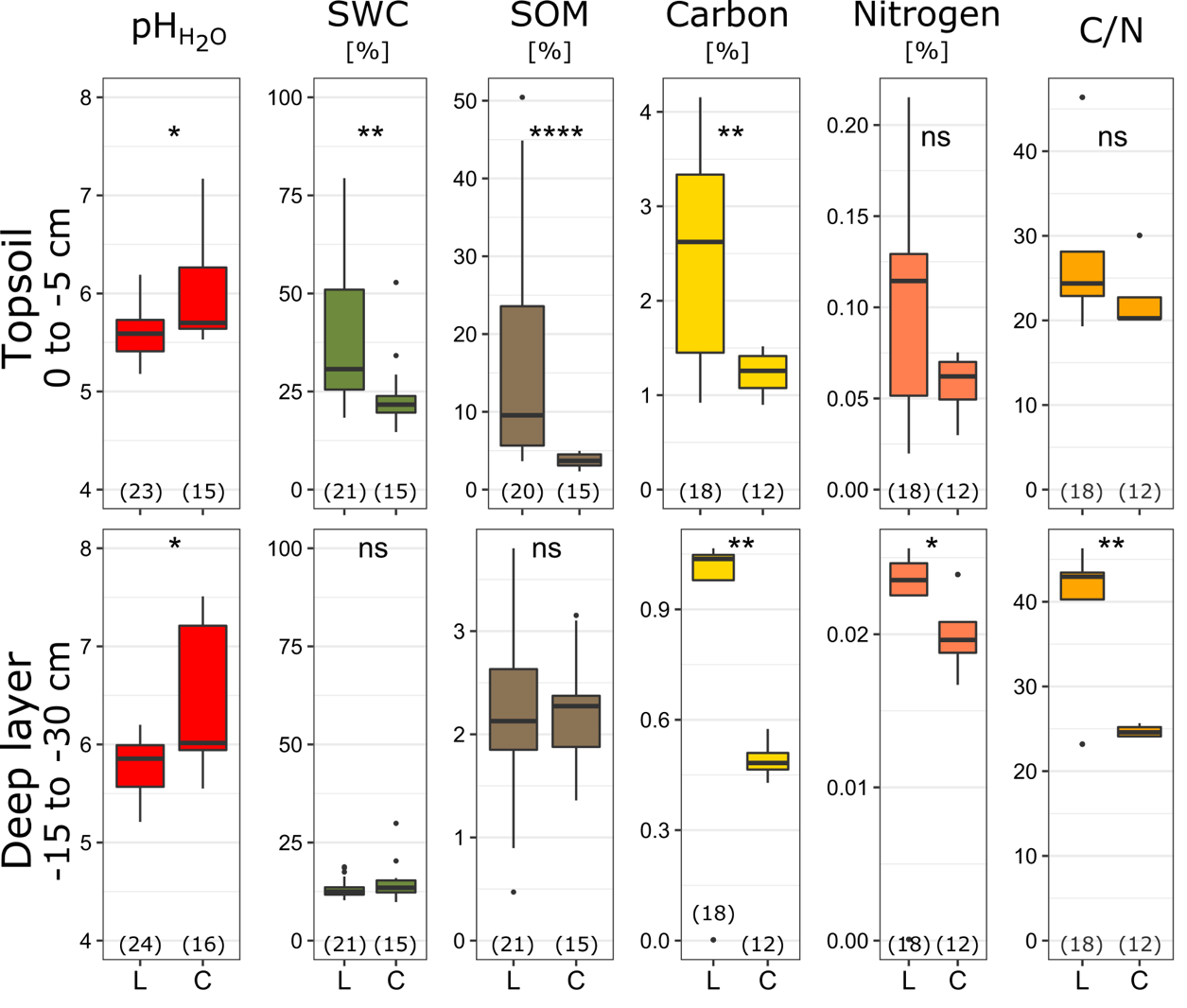


**Fig. S1:** Soil physical chemical parameters measured in topsoil and deep layer samples over the growing season of 2017 at the two studied tussock tundra sites Latnjajaure (L) and Corrvosjávri (C); SWC: gravimetric soil water content; SOM: soil organic matter; C/N: carbon to nitrogen ratio. Difference between group means was tested by a Wilcoxon rank sum test (ns, *, ** and **** = not significant, significant at alpha = 0.05, 0.01 and 0.0001 respectively). Numbers at the bottom of the graph represent the number of observations per group.


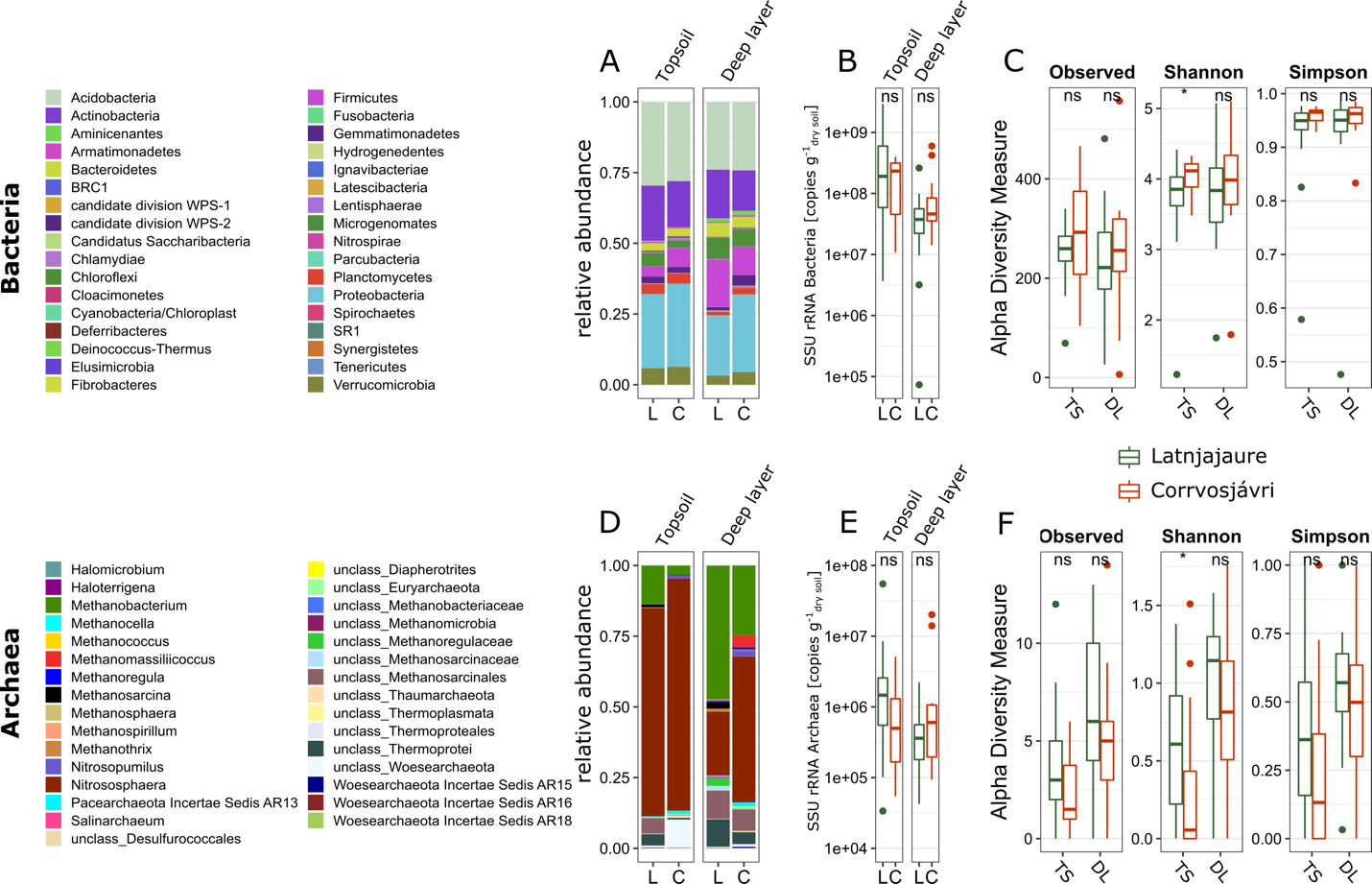


Fig. S2­­­­­­­: Relative (A+D) and absolute abundance (B+E) as well as alpha diversity estimates (C+F) of bacterial (top panels) and archaeal (bottom panels) communities of the two studied tussock tundra sites Latnjajaure (L) and Corrvosjávri (C).TS = topsoil; DL = deep layer. Relative abundances are shown as annotated *16S rRNA* amplicons on phylum and genus level for bacteria and archaea respectively; absolute abundances are show as copy numbers of *16S rRNA* genes estimated by quantitative PCR and alpha diversity estimates were calculated from taxonomic tables of sequenced *16S rRNA* amplicons. Differences between group means of sites were tested by a Wilcoxon rank sum test (ns and * = not significant and significant at alpha = 0.05 respectively).


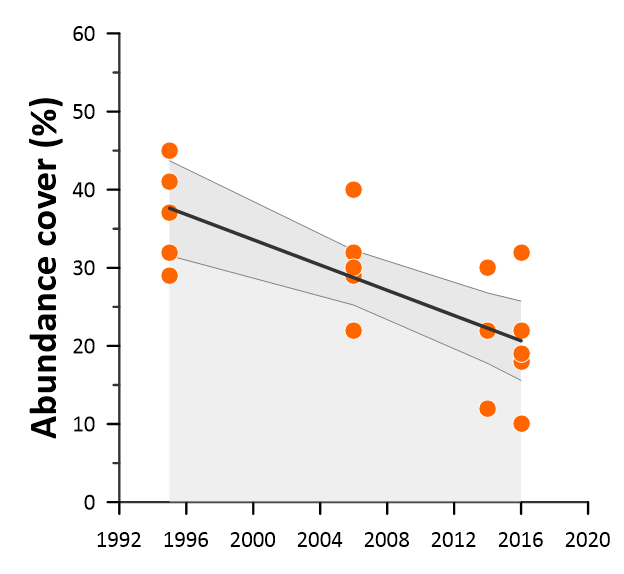


Fig. S3: Abundance cover of *Eriophorum vaginatum* at the Latnjajaure tussock tundra community over time, showing a significant loss 8.1 % per decade (R^2^ = 0.51, p-value < 0.001). Point-intercept data from the five permanent marked control plots within the International Tundra International Tundra Experiment (ITEX) (Molau 2010; Scharn et al. 2021).

Table S1: Interpolated flux of CH_4_ using time point interpolation. Interpolations were done for period start of July to end of August for the growing seasons 2016-2018, the bi-weekly samplings generally started earlier and progressed later but to ensure a similar length for all years this interval was used. An exponential model between CH_4_ emissions and soil temperature was established to estimate seasonal flux and Q_10_ values, while Activation energy (*Ea*) was established from the Arrhenius curve.

|  | Integrated flux  [mg C-CH_4_ m^-2^ period^-1^] | Exponential model  [mg C-CH_4_ m^-2^ period^1^] | *Ea*  [kJ mol^-1^] | Q_10_ |
| --- | --- | --- | --- | --- |
| Latnjajaure | 266.0 ± 40.2*^a^* | 556.5 ± 91.7 | 143.5 ± 1.5 | 8.8 ± 0.2 |
| Corrvosjávri | 8.1 ± 4.5*^a^* | -* | -* | -* |
| ^a^ significant difference between the sites (p-value < 0.001)  * No significant exponential fit could be established for the low Corrvosjávri emission, and thereby *E_a_* or Q_10_ could not be calculated. | | | | |

Table S2: Differential abundance analysis using the R package DESeq2 on annotated *16S rRNA* amplicon sequences. A potential role in the carbon cycle of each taxa was based on a literature search of the respective genus: *syntrophic:* syntrophic relationship with a methane producer has been observed; *potential syntrophic:* fermenting organisms producing precursors for methanogens like acetate, fumarate and hydrogen; *homoacetogenic:* CO_2_ and H_2_ are used to generate acetate; *methanogenic:* organism which actively produces methane; *cellulolytic* and *chitinolytic:* degradation of recalcitrant organic matter observed like cellulose or chitin respectively. Oxygen requirements: A - aerobic; FA - facultative anaerobic; OA - obligate anaerobic.

| **Taxa higher abundant in Latnjajaure** | | | | | |
| --- | --- | --- | --- | --- | --- |
| **Taxa** | **[log2 fold change]** | **p-value** | **Potential role in carbon cycle** | **O_2_ requirements** | **References** |
| Pelotomaculum | 5.4 ± 1.0 | 5.4E-08 | syntrophic | OA | (Imachi et al. 2007; Kouzuma, Kato, and Watanabe 2015) |
| Acetobacterium | 5.1 ± 0.9 | 1.7E-08 | homoacetogenic | OA | (Simankova et al. 2000) |
| Saccharofermentans | 4.9 ± 0.8 | 6.2E-10 | potential syntrophic | OA | (Chen, Niu, and Zhang 2010) |
| Sulfuricella | 4.8 ± 0.9 | 6.0E-08 | - | FA |  |
| Unclassified Methanosarcinales | 4.4 ± 1.0 | 1.4E-05 | methanogenic | OA | (Altshuler, Goordial, and Whyte 2017) |
| Syntrophomonas | 4.3 ± 1.0 | 3.4E-05 | syntrophic | OA | (Mcinerney et al. 1981; Wu, Liu, and Dong 2006) |
| Cryptanaerobacter | 4.2 ± 1.2 | 3.2E-04 | potential syntrophic | OA | (Ahlert et al. 2016) |
| Natranaerovirga | 4.2 ± 1.0 | 4.3E-05 | potential syntrophic | OA | (Sorokin et al. 2012) |
| Syntrophorhabdus | 3.9 ± 1.0 | 5.9E-05 | potential syntrophic | OA | (Qiu et al. 2008) |
| Smithella | 3.9 ± 0.8 | 3.3E-07 | syntrophic | OA | (Liu et al. 1999) |
| Corynebacterium | 3.9 ± 1.2 | 8.3E-04 | - | - |  |
| Methanosarcina | 3.9 ± 1.0 | 1.9E-04 | methanogenic | OA | (Altshuler, Goordial, and Whyte 2017) |
| Demequina | 3.2 ± 0.8 | 7.2E-05 | - | FA | (Ue et al. 2011) |
| Cellulomonas | 3.0 ± 0.5 | 3.4E-08 | cellulolytic | FA | (Christopherson et al. 2013) |
| Methanobacterium | 2.8 ± 0.7 | 1.0E-04 | methanogenic | OA | (Altshuler, Goordial, and Whyte 2017) |
| Rhodoferax | 2.0 ± 0.6 | 6.3E-04 | potential syntrophic | FA | (Finneran, Johnsen, and Lovley 2003) |
| **Taxa higher abundant in Corrvosjávri** | | | | | |
| Terrimonas | 4.4 ± 0.8 | 7.0E-08 | potential chitinolytic | A | (Jiang et al. 2014; Kim et al. 2017) |
| Frondihabitans | 4.1 ± 0.9 | 2.4E-06 | - | A | (Lee 2010) |
| Blastochloris | 3.5 ± 0.6 | 4.0E-10 | - | FA |  |
| Aquaspirillum | 2.8 ± 0.7 | 5.7E-05 | - | A | (Butler, Mccallum, and Inniss 1989) |
| Actinospica | 2.6 ± 0.7 | 6.6E-05 | - | A | (Golinska et al. 2015; Cavaletti et al. 2006) |
| Chitinophaga | 2.6 ± 0.8 | 8.3E-04 | chitinolytic | A | (Sangkhobol and Skerman 1981; Pankratov et al. 2006) |
| Anaeromyxobacter | 2.0 ± 0.4 | 6.2E-07 | potential syntrophic | FA | (Hattori 2008; Sanford, Cole, and Tiedje 2002) |
| Coxiella | 2.0 ± 0.6 | 5.0E-04 | - |  |  |

**Table S3:** Lapse rates at the Abisko region established using the automatic weather station data from Latnjajaure Field Station and the meteorological observations from Abisko Scientific Research Station for the period 1993-2019. As a reference point, environmental lapse rates from a one-year investigation (July 2008–June 2009) by Yang el al. (Yang et al. 2012) is also shown.

|  | This Study | Yang et al., 2012 |
| --- | --- | --- |
| Annual rate | – 3.4 | − 3.8 |
| January | 2.6 | − 1.1 |
| February | 1.4 | 10.4 |
| March | – 2.6 | − 5.8 |
| April | – 5.7 | − 8.7 |
| May | – 6.6 | − 9.2 |
| June | – 7.7 | − 6 |
| July | – 6.0 | *NA* |
| August | – 4.9 | − 6.3 |
| September | – 4.6 | − 5.4 |
| October | – 4.1 | − 3.4 |
| November | – 1.9 | − 2.6 |
| December | – 1.1 | *NA* |

**SI References**

Ahlert, Stephan, Rita Zimmermann, Johannes Ebling, and Helmut König. 2016. “Analysis of Propionate-Degrading Consortia from Agricultural Biogas Plants.” *MicrobiologyOpen* 5 (6): 1027–37. https://doi.org/10.1002/mbo3.386.

Altshuler, Ianina, Jacqueline Goordial, and Lyle G. Whyte. 2017. “Microbial Life in Permafrost.” In *Psychrophiles: From Biodiversity to Biotechnology: Second Edition*, 153–79. https://doi.org/10.1007/978-3-319-57057-0.

Butler, Barbara J., Kirk L. Mccallum, and William E. Inniss. 1989. “Characterization of Aquaspirillum Arcticum Sp. Nov., a New Psychrophilic Bacterium.” *Systematic and Applied Microbiology* 12 (3): 263–66. https://doi.org/10.1016/S0723-2020(89)80072-4.

Cavaletti, Linda, Paolo Monciardini, Peter Schumann, Manfred Rohde, Ruggiero Bamonte, Elena Busti, Margherita Sosio, and Stefano Donadio. 2006. “Actinospica Robiniae Gen. Nov., Sp. Nov. and Actinospica Acidiphila Sp. Nov.: Proposal for Actinospicaceae Fam. Nov. and Catenulisporinae Subord. Nov. in the Order Actinomycetales.” *International Journal of Systematic and Evolutionary Microbiology* 56 (8): 1747–53. https://doi.org/10.1099/ijs.0.63859-0.

Chen, Shuangya, Lili Niu, and Yongxiang Zhang. 2010. “Saccharofermentans Acetigenes Gen. Nov., Sp. Nov., an Anaerobic Bacterium Isolated from Sludge Treating Brewery Wastewater.” *International Journal of Systematic and Evolutionary Microbiology* 60 (12): 2735–38. https://doi.org/10.1099/ijs.0.017590-0.

Christopherson, Melissa R., Garret Suen, Shanti Bramhacharya, Kelsea A. Jewell, Frank O. Aylward, David Mead, and Phillip J. Brumm. 2013. “The Genome Sequences of Cellulomonas Fimi and ‘Cellvibrio Gilvus’ Reveal the Cellulolytic Strategies of Two Facultative Anaerobes, Transfer of ‘Cellvibrio Gilvus’ to the Genus Cellulomonas, and Proposal of Cellulomonas Gilvus Sp. Nov.” *PLoS ONE* 8 (1). https://doi.org/10.1371/journal.pone.0053954.

Finneran, Kevin T., Claudia V. Johnsen, and Derek R. Lovley. 2003. “Rhodoferax Ferrireducens Sp. Nov., a Psychrotolerant, Facultatively Anaerobic Bacterium That Oxidizes Acetate with the Reduction of Fe(III).” *International Journal of Systematic and Evolutionary Microbiology* 53 (3): 669–73. https://doi.org/10.1099/ijs.0.02298-0.

Golinska, Patrycja, Tiago Domingues Zucchi, Leonardo Silva, Hanna Dahm, and Michael Goodfellow. 2015. “Actinospica Durhamensis Sp. Nov., Isolated from a Spruce Forest Soil.” *Antonie van Leeuwenhoek, International Journal of General and Molecular Microbiology* 108 (2): 435–42. https://doi.org/10.1007/s10482-015-0496-1.

Hattori, Satoshi. 2008. “Syntrophic Acetate-Oxidizing Microbes in Methanogenic Environments.” *Microbes and Environments* 23 (2): 118–27. https://doi.org/10.1264/jsme2.23.118.

Imachi, Hiroyuki, Sanae Sakai, Akiyoshi Ohashi, Hideki Harada, Satoshi Hanada, Yoichi Kamagata, and Yuji Sekiguchi. 2007. “Pelotomaculum Propionicium Sp. Nov., an Anaerobic, Mesophilic, Obligately Syntrophic, Propionate-Oxidizing Bacterium.” *International Journal of Systematic and Evolutionary Microbiology* 57 (7): 1487–92. https://doi.org/10.1099/ijs.0.64925-0.

Jiang, Fan, Xia Qiu, Xulu Chang, Zhihao Qu, Lvzhi Ren, Wenjing Kan, Youhao Guo, Chengxiang Fang, and Fang Peng. 2014. “Terrimonas Arctica Sp. Nov., Isolated from Arctic Tundra Soil.” *International Journal of Systematic and Evolutionary Microbiology* 64: 3798–3803. https://doi.org/10.1099/ijs.0.067033-0.

Kim, Myong Chol, Ok Chol Kang, Chol Myong Kim, Yumin Zhang, Zuobing Liu, Ziyan Wei, Yao Huang, Wangmu Danzeng, and Fang Peng. 2017. “Terrimonas Crocea Sp. Nov., Isolated from the till of a High Arctic Glacier.” *International Journal of Systematic and Evolutionary Microbiology* 67 (4): 868–74. https://doi.org/10.1099/ijsem.0.001689.

Kouzuma, Atsushi, Souichiro Kato, and Kazuya Watanabe. 2015. “Microbial Interspecies Interactions: Recent Findings in Syntrophic Consortia.” *Frontiers in Microbiology* 6 (MAY): 1–8. https://doi.org/10.3389/fmicb.2015.00477.

Lee, Soon Dong. 2010. “Frondihabitans Peucedani Sp. Nov., an Actinobacterium Isolated from Rhizosphere Soil, and Emended Description of the Genus Frondihabitans Greene et al. 2009.” *International Journal of Systematic and Evolutionary Microbiology* 60 (8): 1740–44. https://doi.org/10.1099/ijs.0.017947-0.

Liu, Yitai, David L Balkwill, C A Henry, Gwendolyn R Drake, and David R Boone. 1999. “Characterization of the Anaerobic Propionate- Degrading Syntrophs Smithella Propionica.” *International Journal of Systematic Bacteriology* 49 (1 999): 545–56. https://doi.org/10.1099/00207713-49-2-545.

Mcinerney, M J, M P Bryant, R B Hespell, and J W Costerton. 1981. “Syntrophomonas Wolfei Gen. Nov. Sp. Nov., an Anaerobic, Syntrophic, Fatty Acid-Oxidizing Bacterium” 41 (4): 1029–39.

Molau, Ulf. 2010. “Long-Term Impacts of Observed and Induced Climate Change on Tussock Tundra near Its Southern Limit in Northern Sweden.” *Plant Ecology and Diversity* 3 (1): 29–34. https://doi.org/10.1080/17550874.2010.487548.

Pankratov, Timofei A., Irina S. Kulichevskaya, Werner Liesack, and Svetland N. Dedysh. 2006. “Isolation of Aerobic, Gliding, Xylanolytic and Laminarinolytic Bacteria from Acidic Sphagnum Peatlands and Emended Description of Chitinophaga Arvensicola Kämpfer et al. 2006.” *International Journal of Systematic and Evolutionary Microbiology* 56 (12): 2761–64. https://doi.org/10.1099/ijs.0.64451-0.

Qiu, Yan Ling, Satoshi Hanada, Akiyoshi Ohashi, Hideki Harada, Yoichi Kamagata, and Yuji Sekiguchi. 2008. “Syntrophorhabdus Aromaticivorans Gen. Nov., Sp. Nov., the First Cultured Anaerobe Capable of Degrading Phenol to Acetate in Obligate Syntrophic Associations with a Hydrogenotrophic Methanogen.” *Applied and Environmental Microbiology* 74 (7): 2051–58. https://doi.org/10.1128/AEM.02378-07.

Sanford, Robert A., James R. Cole, and James M. Tiedje. 2002. “Characterization and Description of Anaeromyxobacter Dehalogenans Gen. Nov., Sp. Nov., an Aryl-Halorespiring Facultative Anaerobic Myxobacterium.” *Applied and Environmental Microbiology* 68 (2): 893–900. https://doi.org/10.1128/AEM.68.2.893-900.2002.

Sangkhobol, V., and V. B.D. Skerman. 1981. “Chitinophaga, a New Genus of Chitinolytic Myxobacteria.” *International Journal of Systematic Bacteriology* 31 (3): 285–93. https://doi.org/10.1099/00207713-31-3-285.

Scharn, R., C. G. Brachmann, A. Patchett, H. Reese, A. D. Bjorkman, J. M. Alatalo, R. G. Björk, A. K. Jägerbrand, U. Molau, and M. P. Björkman. 2021. “Vegetation Responses to 26 Years of Warming at Latnjajaure Field Station, Northern Sweden.” *Arctic Science*.

Simankova, Maria V., Oleg R. Kotsyurbenko, Erko Stackebrandt, Nadezhda A. Kostrikina, Anatoliy M. Lysenko, Georgiy A. Osipov, and Alla N. Nozhevnikova. 2000. “Acetobacterium Tundrae Sp. Nov., a New Psychrophilic Acetogenic Bacterium from Tundra Soil.” *Archives of Microbiology* 174 (6): 440–47. https://doi.org/10.1007/s002030000229.

Sorokin, D. Y., T. P. Tourova, A. N. Panteleeva, E. N. Kaparullina, and G. Muyzer. 2012. “Anaerobic Utilization of Pectinous Substrates at Extremely Haloalkaline Conditions by Natranaerovirga Pectinivora Gen. Nov., Sp. Nov., and Natranaerovirga Hydrolytica Sp. Nov., Isolated from Hypersaline Soda Lakes.” *Extremophiles* 16 (2): 307–15. https://doi.org/10.1007/s00792-012-0431-6.

Ue, Harumi, Yoshihide Matsuo, Hiroaki Kasai, and Akira Yokota. 2011. “Demequina Globuliformis Sp. Nov., Demequina Oxidasica Sp. Nov. and Demequina Aurantiaca Sp. Nov., Actinobacteria Isolated from Marine Environments, and Proposal of Demequinaceae Fam. Nov.” *International Journal of Systematic and Evolutionary Microbiology* 61 (6): 1322–29. https://doi.org/10.1099/ijs.0.024299-0.

Wu, Chenggang, Xiaoli Liu, and Xiuzhu Dong. 2006. “Syntrophomonas Cellicola Sp. Nov., a Spore-Forming Syntrophic Bacterium Isolated from a Distilled-Spirit-Fermenting Cellar, and Assignment of Syntrophospora Bryantii to Syntrophomonas Bryantii Comb. Nov.” *International Journal of Systematic and Evolutionary Microbiology* 56 (10): 2331–35. https://doi.org/10.1099/ijs.0.64377-0.

Yang, Zhenlin, Edward Hanna, Terry V. Callaghan, and Christer Jonasson. 2012. “How Can Meteorological Observations and Microclimate Simulations Improve Understanding of 1913-2010 Climate Change around Abisko, Swedish Lapland?” *Meteorological Applications* 19 (4): 454–63. https://doi.org/10.1002/met.276.
